# Zinc Oxide Nanoparticles Protected with Terpenoids as a Substance in Redox Imbalance Normalization in Burns

**DOI:** 10.3390/ph14060492

**Published:** 2021-05-21

**Authors:** Nina Melnikova, Alyona Balakireva, Dmitry Orekhov, Denis Kamorin, Natalia Didenko, Darina Malygina, Alexander Knyazev, Denis Novopoltsev, Anna Solovyeva

**Affiliations:** 1 Faculty of Chemistry, Lobachevsky University, 23/5 Gagarin Av., 603950 Nizhny Novgorod, Russia; knyazevav@gmail.com; 2 Engineering-Technology Faculty, Nizhny Novgorod State Technical University n.a. R.E. Alekseev, 24 Minin St., 603950 Nizhny Novgorod, Russia; mitriy07@mail.ru (D.O.); d.kamorin@mail.ru (D.K.); 3 Central Research Laboratory, Privolzhsky Research Medical University, 10/1 Minin Sq., 603950 Nizhny Novgorod, Russia; Liza200000@yandex.ru (A.B.); natalika-nv@mail.ru (N.D.); dennovopoltsev@mail.ru (D.N.); sannag5@mail.ru (A.S.); 4 Department of Pharmaceutical Chemistry, Privolzhsky Research Medical University, 10/1 Minin Sq., 603950 Nizhny Novgorod, Russia; mds73@yandex.ru

**Keywords:** hydro- and oleo-dispersions of zinc oxide nanoparticles, lavender oil, thyme oil, betulin, betulin diphosphate, terpenoids, redox balance of NAD+/NADH and NADP+/NADPH

## Abstract

Preliminary protection of zinc oxide nanoparticles (ZnO NPs) with terpenoids such as betulin, its derivatives, and essential oils components has been proposed to produce gel-like oleophilic and hydrophilic formulations. We studied the properties of gel-like dispersions of ZnO NPs with immobilized terpenoids and their effects on the activity of LDH, GR, G6PDH, restoration of redox balance of co-enzyme pairs NAD+/NADH and NADP+/NADPH, as well as the activity of SOD, catalase, AlDH in erythrocytes in the treatment of burns in rats. Hysteresis loops on the rheograms of studied dispersions characterize their thixotropic properties. ZnO NPs with betulin diphosphate in the water–ethanol medium lead to a 20-fold increase in the hydrodynamic radius at pH 7.3 compared to the initial ZnO NPs, and facilitate the formation of Zn^2+^ ions and their penetration into the viable epidermis, unlike oleophilic dispersions. All dispersions reduce the healing time by one and a half times compared with the untreated control group, increase the activity of LDH, GR, G6PDH, SOD, catalase, AlDH, and contribute to the normalization of coenzyme balance. Normalization of the redox balance and wound state was more effective using hydrophilic dispersions due to Zn^2 +^ penetration.

## 1. Introduction

A burn injury that causes burn disease is accompanied by intense inflammation, tissue damage and infection throughout the body, and a change in the general metabolism [1,2,3,4,5].

At the molecular level, one of the mechanisms for the development of burn disease is the excessive production of cytotoxic reactive oxygen species (ROS), which leads to systemic inflammatory response syndrome (SIRS), sepsis, and multiple organ failure, and is the leading cause of mortality [4,5,6,7,8].

Oxidative stress is counteracted by the enzymatic antioxidant defense system—superoxide dismutase (SOD), catalase, etc. [9,10]. Reducing oxidative stress, i.e., the antioxidant effect, is also promoted by pyridine nucleotides—nicotinamide adenine dinucleotide and its reduced form (NAD+/NADH), nicotinamide adenine dinucleotide phosphate and its reduced form (NADP+/NADPH), participating in the work of some other enzymes, mainly oxidoreductases [11,12,13]. NAD (including NAD+ and NADH) is mainly used by enzymes that catalyze substrate oxidation, while NADP (including NADP+ and NADPH) catalyzes substrate reduction [14].

The regeneration of the balance between the pairs of coenzymes NAD+/NADH and NADP+/NADPH can be seriously impaired during burn disease under hypoxia conditions. A consequence of the redox imbalance is an undesirable change in the levels of lactate dehydrogenase (LDH), glucose-6-phosphate dehydrogenase (G6PD), glutathione reductase (GR), and aldehyde dehydrogenase (AlDH), which also contributes to the detoxification of the malonic dialdehyde (MDA).

On the other hand, it is essential to create factors that promote regeneration and cellular respiration in burn tissues, reduce acidosis and edema, and remove exudate. In local treatment, it is important to make a moist environment in the wound due to hydrophilic external agents [15] or protect the wound surface with oleophilic agents, such as betulin oleogel sunflower oil which promotes better re-epithelialization [16]. The therapeutical effects (reabsorption and penetration of the drug) also depend on the rheological characteristics of topical dosage forms. 

We have previously shown [17] that an oleogel containing zinc oxide nanoparticles (ZnO NPs) and betulin derivatives effectively treats burn wounds and improves overall health due to predominantly activating the antioxidant enzymatic defense mechanism, improving energy metabolism, and reducing MDA levels.

It is desirable to reduce the potential toxicity of ZnO NPs in the human body under systemic exposure due to the possibility of forming rigid protein crowns on the surfaces of nanoparticles in a body [18,19,20,21,22]. Traditionally, the protection of ZnO NPs is functionalization with protein repellent molecules such as PEG or phosphoric polyesters [23,24], which decrease binding to surface proteins.

Early we used the protection of the ZnO NPs surface by pretreating nanoparticles with ethanolic solutions of betulin derivatives, capable of binding zinc ions on the surface of ZnO NPs and reducing the formation of protein crowns. These protected ZnO NPs have been used as components of anti-burn wound dressings and oleogels [17,25]. Despite the effectiveness of ZnO NP oleogels with impregnated triterpenoids, the application of these dosage forms in practice has some limitations. This is due to the low stability of oleogels, fluidity of oleogels on the wound surface, absorption of a part of the drug into the dressing material, as well as penetration only into the stratum corneum without reaching the viable epidermis.

In this work, we studied the physicochemical properties of anti-burn hydrophilic and oleophilic gel-like dispersions of ZnO NPs protected by terpenoids and their effect on the biochemical parameters in an experiment on rats. We evaluated the activity of enzymes LDH, G6PDG, AlDH, GR, determined by the balance of coenzymes NAD+/NADH and NADP+/NADPH, and SOD and catalase, and the level of MDA. 

**Figure 1:**
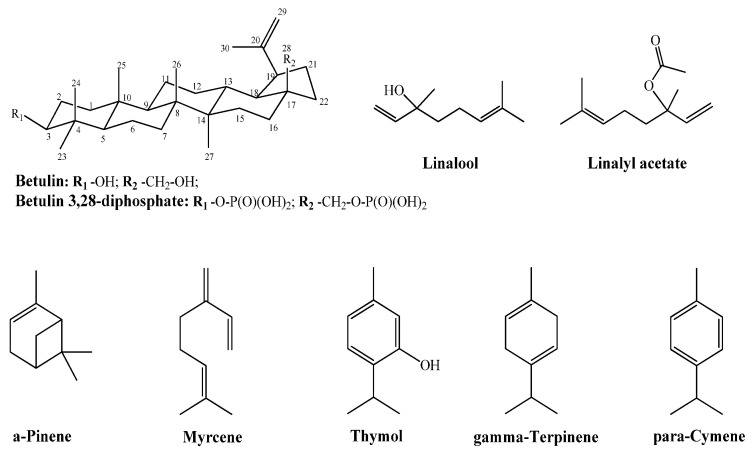
Formulas of the studied terpenoids.

As the best results for the use of oleogels were found for BDP-containing ones, we used ZnO NPs protected by betulin (B) or betulin diphosphate (BDP) in the presence of antioxidant and anti-inflammatory terpenoids of lavender and thyme oil, as well as their natural component, for example, thymol (Figure 1).

## 2. Results

### 2.1. Characterization of Zinc Oxide Nanoparticles

Data of UV, FTIR, PL spectrophotometries, and PXRD of initial ZnO NPs samples are typical for ZnO NPs (Figure S1, Supplementary Materials).

PXRD pattern corresponds to hexagonal wurtzite structure with average diameter equaled to 12–18 nm, calculated using Scherrer’s equation (*n* = 3). There is an intense band near 468 cm^−1^ of FTIR spectra and a band of exciton absorption of UV-spectrum in the region of 345–360 nm. The PL-spectrum of ZnO NPs dispersion contains a sharp blue emission absorption band λ_em_ = 372 nm and a shoulder at 400–420 nm, which characterizes the edge emission of quantum dots and the onset of ultraviolet absorption. SEM images (Figure S2a) of ZnO NPs demonstrate light amorphous powder in the absence of ZnO NPs aggregation. The specific surface area of ZnO NPs without terpenes protection is equal to 68.0 ± 0.8 m^2^·g^−1^ (*n* = 3).

The assay of ZnO NPs was carried out by atomic absorption spectrophotometry. The impurities (lithium, sodium, magnesium, calcium, iron) as 10–20 ppm were estimated by ICP methods.

### 2.2. Properties of Modified ZnO NPs as a Component of Gel-Like Dispersions

ZnO NPs were treated by essential oils of ethanol solutions or BDP water–ethanol (1:1) solutions previously to decrease possible aggregation.

The specific surface area of ZnO NPs with terpenes protection is equal to 52.7 ± 1.1 m^2^·g^−1^, which characterizes terpenes immobilization onto ZnO NPs surface.

**Figure 2:**
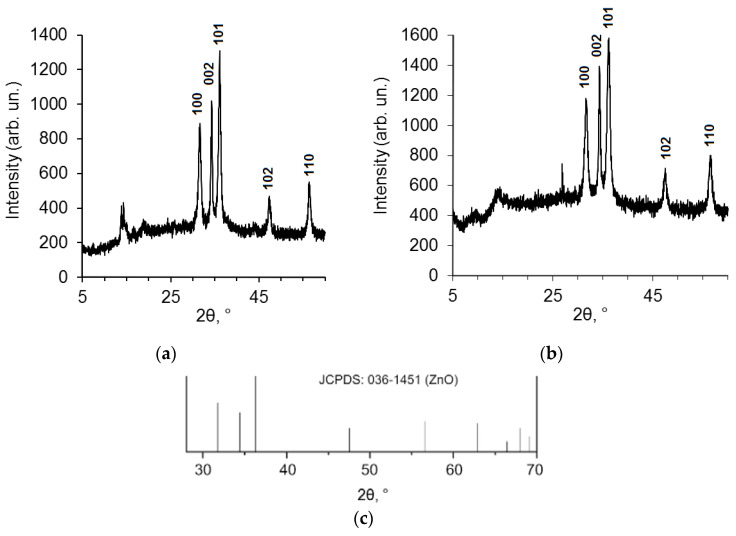
PXRD patterns of ZnO NPs modified by lavender oil (**a**) and BDP (**b**) taken from [17]; standard JCPDS pattern for ZnO, file 036-1451 (**c**).

Figure S2b shows that the SEM image of ZnO NPs modified by BDP (ZnO NPs-BDP) is similar to the SEM image of ZnO NPs. Nanoparticles sizes of ZnO NPs calculated by PXRD are close to ZnO NPs-BDP and ZnO NPs-lavender oil sizes. This fact characterizes the absence of nanoparticle aggregation after modification (Figure 2, Table 1).

XRD peak appearance at 10° for ZnO NPs in lavender oil absent in standard JCPDS pattern for ZnO (Figure 2c) possibly belongs to terpenes in lavender oil. For example, natural borneol has a signal at 8–15° in XRD patterns [26]. A sharp peak at around 28° in Figure 2b possibly belongs to one of the BDP polymorphs adsorbed on the surface of the ZnO NPs [27].

FTIR-spectra of ZnO NPs modified by betulin and BDP suggest the inclusion of triterpenoid molecules into ZnO NPs (Figure S3).

To choose the conditions for the ZnO NPs stabilization, we carried out experiments to assess the zeta potential of dispersions containing BDP or citric acid as a control to compare the effectiveness of ZnO NPs stabilizers [28]. Since BDP, unlike citric acid, is slightly soluble in water, comparative studies were carried out in an aqueous ethanol medium at a relatively low concentration of the studied organic substances. Table 2 shows that the zeta potential of ZnO NPs, both in an aqueous ethanol medium of citric acid and in BDP at pH 7.3, has low values and does not provide a high stability of dispersions. Zeta potential of the more polar sodium salt of BDP has a close value to the zeta potential of BDP, which is caused by the existence of BDP as its salt at pH 7.3.

Taking these data into account, in this study and the previous one [25], we used ZnO NPs pretreated with a 0.1 M ethanolic solution of BDP. The zeta potential of ZnO NPs-BDP in an ethanol solution of BDP, equal to −41.20 mV, meets the requirements for the stability of dispersions of pre-modified ZnO NPs.

**Figure 3:**
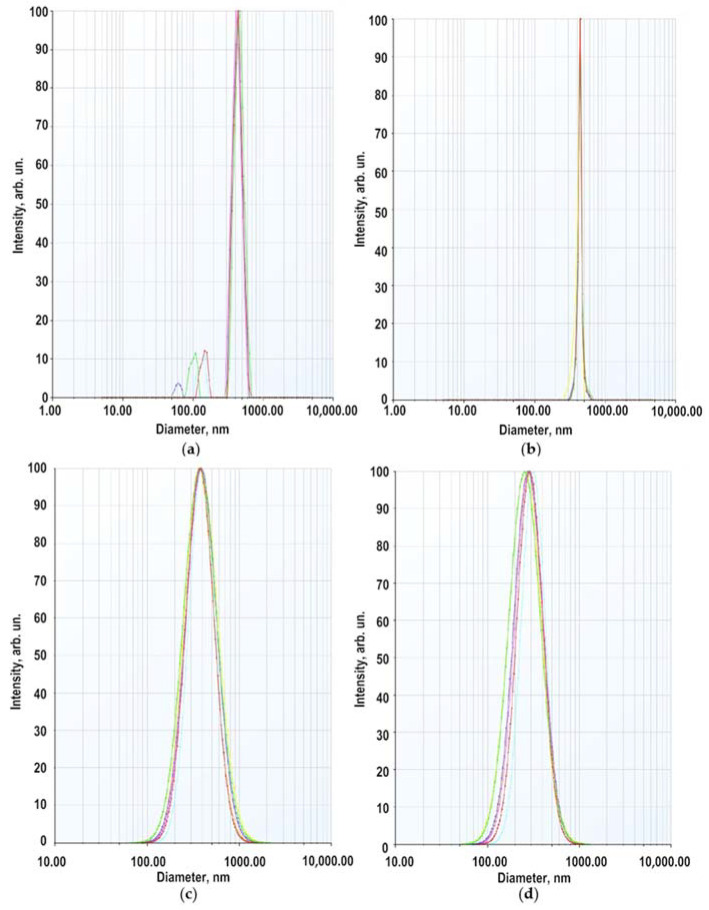
Size distribution of ZnO NPs in 5 × 10^−4^ M sodium salt of BDP (ethanol: water 1:1): (**a**) by scattering intensity (multimodal regime); (**b**) by volume of dispersion (multimodal regime); (**c**) by scattering intensity (lognormal mode); (**d**) by volume of scattering (lognormal mode).

The secondary size (hydrodynamic radius) distribution measurement of ZnO NPs dispersion in a 5 × 10^−4^ M BDP in ethanol: water (1:1) at pH 7.3 was carried out by the dynamic light scattering (DLS) method. Two modes were recorded in the multimodal regime with maxima at 60–150 nm and 350–450 nm (Figure 3a). The weight (volume) distribution contained one mode with a maximum at 440 nm (Figure 3b). In the lognormal model, the intensity-averaged diameter (on all particles) = 380 nm, the volume-averaged diameter 278 nm (Figure 3c,d).

The average hydrodynamic diameter of ZnO NPs in BDP water–ethanol media indicates particle aggregation. The hydrodynamic sizes and zeta potential values are in good agreement with a study on the agglomeration of ZnO NPs in aqueous media of citric acid at pH 7.5 [28]. In studied dispersions, the number of collisions due to inter-particular interactions increases whereas, the path average length traveled by particles between successive collisions falls. We suppose that increasing ZnONP size (380 nm by the intensity and 278 nm by volume) is due to soft corona formation composed of BDP and its sodium salt at pH 7.3. Hence, dispersed ZnO NPs exhibited a hydrated surface wrapped within a cover of molecules not including the ingredients of ZnO NPs themselves.

Therefore, the higher value of average size obtained in DLS measurement compared to the PXRD and SEM analysis was observed possibly due to the (i) agglomeration of ZnO NPs-BDP and (ii) DLS measures the hydrodynamic radii of the nanoparticles, which includes the solvent layer at the interface. 

The role of phosphate groups of ionized BDP in ZnO NPs capping and stabilization was clearly evident. This result differs from data of betulin immobilized at the metallic surface such as gold and silver nanoparticles [29,30]. In this case, for stabilization of aqueous dispersion, it is necessary to use polar PEG or PEG-SH covalently bonding to the metallic surface as a coating agent. DLS measurement showed an increase in the average size of silver and gold nanoparticles from 14–15 nm to 70–75 nm.

Thus, the strong increase in ZnO NPs-BDP hydrodynamic radii in polar medium up to 350–450 nm is a good argument for developing hydrophilic dosage forms.

### 2.3. Preparation of Zinc Oxide Nanoparticles with Essential Oils and Triterpenoids

Another approach by creating dosage forms containing low-soluble betulin derivatives is forming inclusion complexes formed by small molecules incorporated into betulin dimers.

The choice of lavender and thyme oils, which have low molecular weight components (Figure 1), is also due to their ability to improve the betulin solubility and its derivatives with a dimeric structure in hydrophilic and lipophilic media.

The essential oil of *Lavandula angustifolia* was analyzed only with the input control for the active ingredients such as linalool (19.98%) and linalyl acetate (19.89%) [31,32]. The composition of lavender oil, according to GLC data, is shown in Figure S4. The composition of thyme oil is presented in Figure S5. The main active pharmaceutical ingredient (API) in thyme oil with antioxidant and anti-inflammatory effects is thymol, although ortho-cymene and linalool make up a significant proportion of thyme oil [33].

We have studied the interaction of thymol as API and betulin in the most detail. Samples of triterpenoids and essential oils were preliminarily ground in the presence of ethanol (colloidal-mechanical treatment). Betulin and its derivatives can form head-to-tail and head-to-head dimeric structures due to hydrophobic binding [34]. As a result of this interaction, cavities are formed inside the dimeric or tetrameric structures, which can accommodate small molecules. Figure S6 shows dimeric or tetrameric structures formed by BDP [35].

The size of the cavity of the betulin derivatives dimers (in diameter), calculated based on quantum-chemical data, allows a smaller molecule, for example, thymol (the topological polar surface area is equal to 20.2 Å^2^), to fit in it (the minimal sizes of the cavity are 6.0–6.7 Å vertically, and 3.2–3.8 Å horizontally).

**Figure 4:**
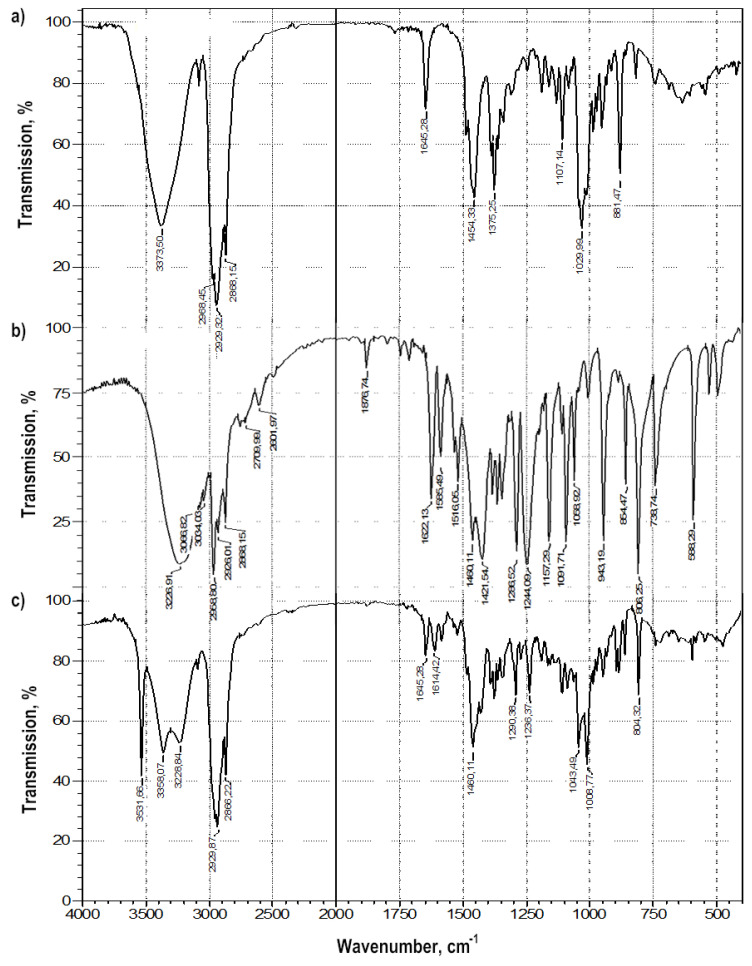
FTIR spectra of: (**a**) betulin, (**b**) thymol, (**c**) the betulin-thymol mixture in a molar ratio of 1:1 after grinding.

Colloidal-mechanical action on a mixture of thymol and betulin led to a change in their FTIR spectra: in the region of the stretching vibration band of the terminal methylene group -C = CH_2_ of betulin (ν_C = C_ 1646 cm^−1^) and the band in the region of 881 cm^−1^ (δCH_2_) (Figure 4). The appearance of a very narrow, intense band in the FTIR spectrum, characteristic of stretching vibrations of alcohol groups O-H, with ν = 3550 cm^−1^, can characterize the emergence of a strong intermolecular hydrogen bond both in the betulin dimer and a π-hydrogen bond.

In general, changes in the bands of stretching vibrations of the aromatic cycle of phenol in the range of 1600–1500 cm^−1^, alcohol and phenolic hydroxyl in betulin and thymol, respectively, are characteristic of molecular associates such as complexes of inclusion of thymol in the betulin dimer (Table 3).

We can assume that the terpene components of lavender oil will also improve the solubility of triterpenoids (B and BDP). Using betulin as an example, an improvement in solubility in an aqueous dispersion medium and a mixture of sunflower and lavender oil or in oils in the presence of thymol was shown in combination with colloidal-mechanical action of almost 1000 times from (1–2) × 10^−6^ mg·mL^−1^ to (1–2) × 10^−3^ mg·mL^−1^.

The immobilization of lower molecular weight components in the surface layers of ZnO NPs was studied in more detail on lavender and thyme oils and thymol with proven pharmacological properties as the main component of thyme oil.

In the systems under study, ZnO NPs particles with adsorbed terpenoids can be considered an independent API since the impregnation of the obtained ZnO NPs with alcoholic solutions of terpenoids is carried out in advance.

The absorption of ZnO NPs in UV spectra changed under modification: the band wavelength in the region of the surface Plasmon resonance spectrum slightly increased from 345 nm (initial ZnO NPs) to 354–360 nm (ZnO NPs-BDP). At the same time, the absorption intensity practically did not change in aqueous-alcoholic dispersions of essential oils and decreased in the presence of betulin and BDP (Figure S7).

**Figure 5:**
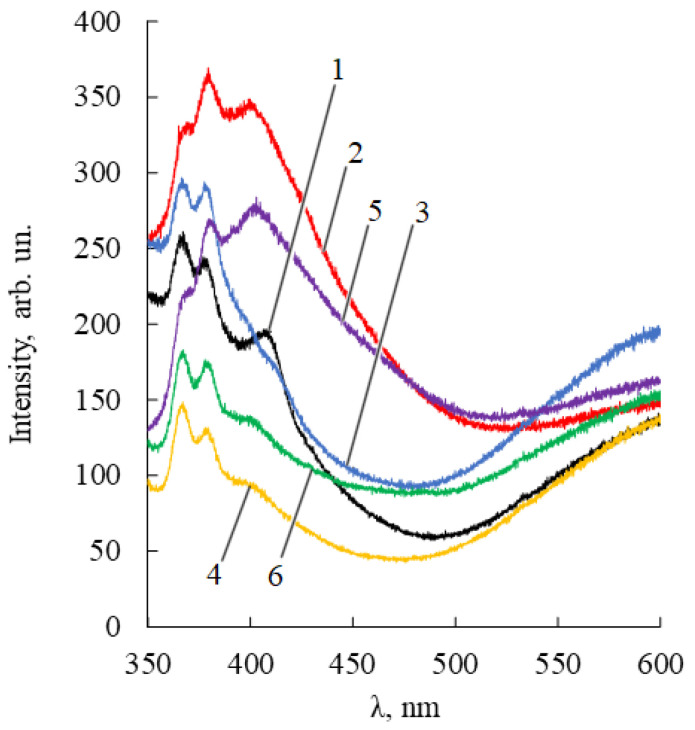
Fluorescence spectra of samples of ZnO NPs dispersions (50 mg/%) in ethanol 95%. ZnO NPs were pretreated with alcohol solutions of 1.4% thymol (1, black); 1.4% thymol and 0.22% betulin (2, red); 1.4% thymol and 0.30% betulin diphosphate (3, blue); 2% lavender oil (4, yellow); 2% lavender oil and 0.22% betulin (5, purple); 2% lavender oil and 0.30% betulin diphosphate (6, green).

Nanoparticles modified by terpenoids were also analyzed by fluorescence spectrophotometry (Figure 5).

The PL ZnO NPs-terpenoids spectra have two emission bands. One of them is in the blue-violet region (360–420 nm) and arises from zinc interstitial defects, which corresponds to exciton radiation in the near-field zone [36]. The second emission band, located in the 420–650 nm region, is due to singly ionized oxygen vacancies (surface defects). The more pronounced the polar nature of adsorbed terpenoids (for example, BDP), the stronger the possibility of their interaction with zinc ions. Accordingly, the more intense the manifestation of the band in the orange region of the PL spectrum.

**Figure 6:**
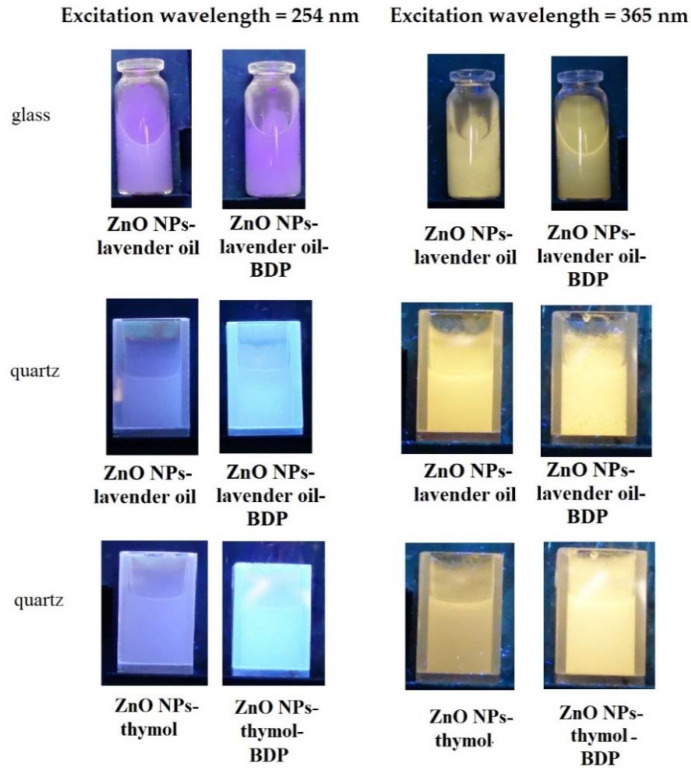
The emission of the pure ZnO NPs and ZnO NPs with lavender oil and thymol in glass vessels or quartz cuvettes under a UV-lamp using excitation wavelengths 254 nm and 365 nm.

The emission of the pure ZnO NPs and ZnO NPs with some terpenoids in glass vessels or quartz cuvettes under a UV-lamp using excitation wavelengths 254 nm and 365 nm are shown in Figure 6.

In general, PL spectra are not only a physicochemical characteristic of ZnO NPs-terpenoids but also indirectly characterize the ability to generate ROS, which is an important property of nanoparticles when interacting with biological membranes [37].

### 2.4. Composition and Properties of Gels

Table 4 shows the compositions of oleophilic and hydrophilic gel-like dispersions of ZnO NPs with immobilized terpenoids developed by us.

The identification and quantification of triterpenoids were performed by HPLC (Figure S8); thymol and lavender oil components were analyzed by GLC. The ZnO NPs protected by terpenes had a PXRD hexagonal wurtzite structure. A typical PXRD pattern is shown in Figure 2. The concentration of ZnO NPs after adsorption of terpenoids in dispersions was estimated by complexometric titration and atomic absorption spectrophotometry (Table 5).

In samples 2 and 3, thymol was introduced into the composition of the dosage form as a natural component of lavender oil and other essential oils, exhibiting anti-inflammatory and antioxidant properties [33].

We prepared gel-like hydrophilic dispersions of B and BDP by preparing antihistamine dosage forms [38] and cosmetic compositions [39]. Surfactants—PEG-30, glyceryl stearate (GS) in the fat phase, and hydroxyethyl cellulose (HEC) in the aqueous phase—were used as structuring components. At the final stage, we added a fatty phase containing terpenoids and ZnO NPs with vigorous stirring to the aqueous phase.

Figure S9 shows digital images of gel-like dispersions (Oleo ZnO NPs-BDP–thymol-lavender and Hydro ZnO NPs-BDP–lavender) under daylight and UV-lamp (λ_ex_ = 254 nm). UV-blue luminescence of Hydro ZnO NPs-BDP–lavender was more intensive than Oleo ZnO NPs-BDP–thymol-lavender.

**Figure 7:**
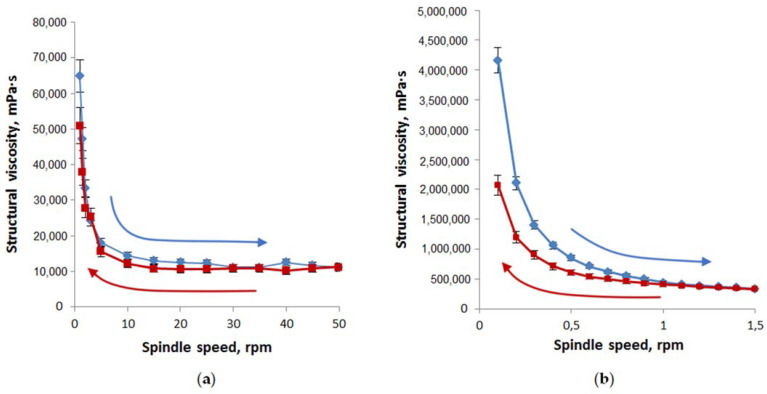
Structural viscosity as a function of spindle speed for oleophilic (**a**) and hydrophilic (**b**) gel-like dispersions (n = 3). Compositions of studied oleophilic and hydrophilic dispersions correspond to samples 2 and 5 in Table 4. Forward rheograms are blue; backward rheograms are red.

Rheological properties were evaluated by the dependence of structural viscosity vs. spindle speed (Figure 7). The thixotropic characters of the designed oleophilic and hydrophilic gel-like dispersions at 25 °C were emphasized by the recorded forward and backward rheograms.

The area values under the curves of structural viscosity in increasing the spindle speed and returning to the initial state showed that the thixotropy of oleophilic dispersions (loop area is equal to 79,470) is significantly less than hydrophilic ones (loop area is equal to 359,480).

We can assume that the rheological properties of the studied gel-like dispersions 2–5, especially hydrophilic ones, will be more convenient dosage forms than oleogel 1 in the treatment of large-area burns.

### 2.5. Characteristics of Animals’ Health State

After the burn during the first three days, the animals’ behavior was calm, and after, up to 9 days later, the animals showed anxiety, indicating pain. On the 10th day, the behavior of the animals was calm while changing the dressings. The wound area was markedly reduced (Table 6).

In general, rat wound healing was more successful with treatment by gel-like dispersions 2–5 than oleogel 1 (Table 4).

On the third day of the experiment, we observed similar histomorphological images of animals treated by Oleo ZnO NPs-BDP–thymol-lavender and Hydro ZnO NPs-BDP–lavender dispersions (Figures S10 and S11). We detected the destruction of the structure of the epidermal tissue upper layers. Coagulation necrosis encompasses both papillary and reticular dermal tissue layers.

On the 10th day of treatment by Oleo ZnO NPs-BDP-thymol-lavender, a wide band of granulation tissue with thin multidirectional collagen fibers was revealed. Macrophages, lymphocytes, fibroblastic cells of varying degrees of maturity, and large, poorly differentiated cells were observed between collagen fibers. The treatment by Hydro ZnO NPs-BDP-lavender led to filling the bottom of the burn wound by granulations and the beginning of connective tissue formation. In control of untreated rats, the epithelial layer of the skin was not restored, even on the 10th day (Figures S10 and S11).

By the 21st day of the experiment, the Hydro ZnO NPs-BDP-lavender group showed granulation tissue maturation and epithelialization signs. Partial peripheral epithelialization can be traced in the Oleo ZnO NPs-BDP–thymol-lavender group (Figures S10 and S11).

The reparation proceeds more intensively with Hydro ZnO NPs-BDP-lavender than with the treatment by Oleo ZnO NPs-BDP-thymol-lavender on the 21st day. This is probably due to the limitation of the destructive process within the epidermis and dermis in a hydrophilic medium under the additional action of the formed zinc ions and lavender oil components.

### 2.6. Evaluation of Energy Metabolism by the Activity of Oxidoreductases Lactate Dehydrogenase and Glucose-6-Phosphate Dehydrogenase in the Treatment of Burn Wounds with Gels-like Dispersions with Zinc Oxide Nanoparticles in Rats

**Figure 8:**
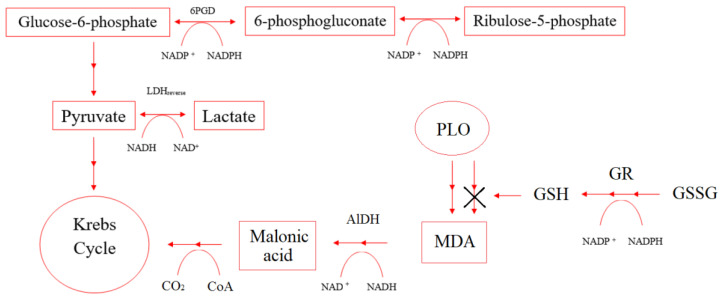
Scheme of NAD+/NADH and NADP+/NADPH participation in some enzymes’ reactions.

Under hypoxia conditions, the imbalance of concentrations in the pairs of the coenzymes NAD+/NADH and NADP+/NADPH affects the enzymes that control cellular respiration and energy metabolism. Figure 8 shows NAD+/NADH and NADP+/NADPH participation in some enzymes’ reactions.

The enhancement of energy metabolism under the action of the studied dosage forms in a burn wound is due to the activation of glucose-6-phosphate dehydrogenase, which is one of the key enzymes of the pentose phosphate pathway. There was an increase in the level of G6PDG compared with the control on day 21 by 38–87% (Table 7). An important function of the enzyme is the formation of cellular NADPH from NADP+, which is necessary to maintain the level of reduced glutathione in the cell, the synthesis of fatty acids, and isoprenoids.

Healthy cells with sufficient oxygen supply use glucose to synthesize carbon dioxide and water until hypoxia occurs. In a burn wound, when blood microcirculation is disturbed in erythrocytes and tissues, oxygen is insufficient. An alternative pathway of ATP synthesis from glucose is the formation of lactate by the mechanism of ineffective anaerobic glycolysis with the participation of LDH.

L-lactate dehydrogenase converts lactate to pyruvate in a direct reaction, which is further used in the Krebs cycle. NAD+ is regenerated by converting pyruvate to lactate (reverse reaction), which characterizes the severity of the cell’s anaerobic process.

Studies have shown that the specific activity of LDH in the direct and reverse reactions decreases after a burn without treatment on days 3 and 7 and slightly increases on day 10 (by 2–34%). When treated with oleogel-like dispersions, the increase in LDH activity in the direct and reverse reactions was 20–40% during the course of treatment. In contrast to oleogels, when treated with hydrophilic drugs, the LDH activity in the reverse reaction increased significantly (by 72% on day 3 and by 53% on day 7). On days 10 and 21, the level of LDH in the reverse reaction returned to normal, while in the direct reaction, LDH activity increased by 18–34% (Table 8).

The glutathione reductase activity increased under the action of the studied drugs (Oleogel ZnO NPs-BDP - lavender, Hydrogel ZnO NPs-BDP) (Table 9). It was due to the regulation of coenzymes NADP+/NADPH and glutathione reduction.

### 2.7. MDA Level Analysis

Markers of the development of oxidative stress resulting from redox imbalance are the level of malondialdehyde (MDA) in blood plasma and erythrocytes (Table 10).

Our results showed that treating a burn wound with the studied dosage forms reduces the MDA level, which characterizes oxidative stress.

### 2.8. The Level of Antioxidant Enzymes (SOD, Catalase)

The data in Table 11 and Table 12 show that the studied dosage forms exhibit antioxidant properties. On day 21, SOD and catalase activities increase by 13–26% and 18–21%, consequently, compared with the norm.

Antioxidant properties improved because AlDH-specific activity increased compared to burned animals without treatment during the initial treatment period. The decrease in oxidative stress and MDA level is evidenced by returning to the normal AlDH level on day 21 of treatment (Table 13).

## 3. Discussion

The data obtained confirm that under hypoxia conditions during thermal injury, the concentration ratio in pairs NADP+/NADPH (G6PDH, GR) and NAD+/NADH (LDH, ADH) changed, which, in turn, are an important cellular redox buffer. It is important to note that in this case, NADPH acts as an antioxidant since it is a coenzyme of GR. Simultaneously, it can act as a direct antioxidant, reducing the action of various free radicals formed during oxidative stress due to the produced glutathione. Besides, it can protect other antioxidant enzymes from ROS-induced inactivation. In addition to its central role in maintaining pools of free glutathione, as mentioned above, NADPH is a major electron donor for reactions catalyzed by cytochrome P450 oxidoreductase. P450 catalyzed monooxygenation of a huge variety of substrates, leading to the addition of one or more hydroxyl groups. It is the cornerstone of phase I detoxification and eliminating most xenobiotics and a wide range of endogenous compounds arising in burn patients under hypoxic conditions.

Enhancement of the wound healing effect and improvement of blood biochemical parameters in treating burn wounds with the studied drugs is probably due to the complex synergistic effect of triterpenoids and lavender and thyme oil components in combination with ZnO NPs.

The effect of ZnO NPs, temporarily and moderately promoting the generation of reactive oxygen species, on the antioxidant properties of the studied dispersions is ambiguous. Thus, in [40], the authors showed that moderate and temporary generation of ROS promotes a decrease in the area of damaged skin in burns, accelerates wound healing, and even stimulates hair growth. Possibly, the contribution of activation of ROS generation has a positive effect under the action of the studied gel-like dispersions of ZnO NPs.

The action of zinc ions, generated by ZnO NPs, does not change the oxidation state in oxidoreductases (LDH, SOD, etc.) and allows us to consider them as an “indirect” antioxidant—a modifier of biological redox reactions. The significant effect of ZnO NPs on the activity of SOD can be partially explained by the activation of the zinc ion in CuZn-SOD, which has extremely high stability and high activity of superoxide dismutation.

On the other hand, zinc’s antioxidant effect is mainly due to the ability to induce a stress response in terms of (1) stimulation of MTF-1-dependent transcription and (2) activation of stress-sensitive signaling cascades MAPK and PI3K/Akt. Moreover, zinc’s antioxidant effect is associated with stabilizing protein thiols (enzymes, zinc fingers, metallothioneins) [41].

The antioxidant action of zinc, in the case of metallothioneins, is to regulate their metabolism. In turn, zinc deficiency leads to a decrease in the protection of sulfhydryl groups and an increase in reactive oxygen species production (ROS). Excessive levels can act as prooxidants, causing a decrease in the level of CuZn-SOD and other important metalloenzymes in erythrocytes. In general, biochemical studies of many diseases, such as diabetes mellitus, have confirmed that zinc’s optimal level is a prerequisite for maintaining normal oxidative metabolism [42].

AlDH is an enzyme that catalyzes the oxidation of a wide range of endogenous and exogenous aldehydes to their corresponding carboxylic acids; it also contributes to the fight against endogenous intoxication syndrome [43,44,45,46]. In particular, the enzyme utilizes aldehydes arising during LPO, thereby reducing the effects of hypoxia. Catalytic reactions of AlDH produce a huge amount of cytosolic NADH as a source of ATP production and play a role in forming acetic acid from acetaldehyde during glycolysis and gluconeogenesis, as well as in the metabolism of amino acids, exogenous chemicals through cytochrome P450, and lipid peroxidase product. The antioxidant effect of aldehyde dehydrogenase concerning the products of free radical oxidation can be noted.

Our results on the increase in GR activity, leading to an increase in glutathione and a simultaneous decrease in MDA levels under the influence of ZnO NPs, are in good agreement with the data obtained by the authors [47].

In general, comparing the activity of SOD, catalase, GR, G6PDH, and LDH enzymes in the treatment of burns in rats, we can note a better normalization of the redox balance of coenzyme pairs NAD+/NADH and NADP+/NADPH by the action of hydrophilic gel-like dispersions (Hydro ZnO NPs-B–lavender and Hydro ZnO NPs-BDP–lavender) compared with oleophilic dispersions.

The increase in the redox activity of the coenzyme pairs NAD+/NADH and NADP+/NADPH and, accordingly, the activity of enzymes in the treatment with hydrophilic gel-like dispersions is due to the possibility of the formation of zinc ions from ZnO NPs in a weakly acidic polar medium created by BDP and their penetration into the skin.

ZnO_(s)_ + 2H^+^_(aq)_ → Zn^2+^_(aq)_ + H_2_O

The relative penetration and permeability of zinc oxide and zinc ions into human skin after applying various formulations with zinc oxide are discussed in [48]. In a hydrophilic environment at pH 6, unlike oil formulations, zinc oxide on the skin surface generates zinc ions in the stratum corneum, hence in the viable epidermis and subsequently systematic circulation.

The effect of zinc ions on the regulation of enzyme activity and the healing of burn wounds is well known. In practice, zinc supplementation is used as an initial therapy for burns [49].

The advantages of using thixotropic gel-like dispersions of ZnO NPs-BDP in comparison with hydrophilic wound dressings based on bacterial cellulose and ZnO NPs with impregnated BDP [25] are the possibility of their use for the treatment of (i) large-area burns; (ii) burns with a complex relief and (iii) burns in hard-to-reach places.

## 4. Materials and Methods

### 4.1. Materials

Lavender oil (CAS 8000-28-0) and thymol (CAS 89-83-8) were purchased from Sigma-Aldrich (Moscow, Russia) without purification. Properties of lavender oil are: boiling point 204 °C, density 0.879 g/cm^3^, refractive index 1.461, acid number no more than 0.8 [31,32].

PEG-30 dipolyhydroxystearate (BASF, Ludwigshafen am Rhein, Germany) and glyceryl stearate (BASF, Ludwigshafen am Rhein, Germany) were used as surfactants. Hydroxyethyl-cellulose (CAS 9004-62-0) was purchased from Sigma-Aldrich (Moscow, Russia).

Betulin was purchased from Sigma-Aldrich (Moscow, Russia, CAS 473-98-3). FTIR, ν, cm^−1^: 3470 st (OH), 1640 st (C = C); ^1^H-NMR δ, ppm: 4.67 m (1H, =CH_2_), 4.57 m (1H, =CH_2_), 3.78 br. s (1H, 28-CH_2_OH), 3.31 m (1H, 28-CH_2_OH), 3.17 m (1H, 3-CHOH), 2.36 m (1H, 19-CH), 1.66 s (3H, CH_3_), 1.23 s (3H, CH_3_), 0.96 s (3H, CH_3_), 0.94 s (3H, CH_3_), 0.80 s (3H, CH_3_), 0.74 s (3H, CH_3_). ^13^C-NMR, δ, ppm: 76.71 (C-3), 109.46 (C-29), 150.24 (C-20), 57.87 (C-28). FTIR-spectral data are presented in Figure S3a.

Betulin-3,28-diphosphate (BDP, 3β,28-diphosphate-lup-20(29)-ene) was synthesized according to the procedure [27].

### 4.2. Synthesis and Characterization of Zinc Oxide Nanoparticles

Synthesis of zinc oxide nanoparticles was carried out in accordance with methods used previously [50,51]. We used freshly prepared 2% solution of sodium hydroxide (or lithium hydroxide) in 96% ethanol (or methanol), and 1.5% solution of zinc acetate dehydrate in 96% ethanol (or methanol) at 70 °C. The solution of sodium or lithium hydroxide (10 mL) was added drop by drop to 30 mL solution of zinc acetate dehydrate in an ice bath and was mixed up for 5–10 min. White flakes generated were precipitated by heptane (60 mL). After the liquid and solid phase separation, the precipitate was washed on a paper filter successively by ethanol and heptane to remove an excess of acetate and sodium ions. The precipitate was dried at 105 ± 5 °C for 5 h.

FTIR spectrum (KBr pellets, IR Prestige-21 FTIR spectrometer, Shimadzu, Kyoto, Japan) has a band in the region 450 cm^−1^ (νZnO) (Figure S1b), UV–vis spectrum in ethanol solution has band in the region 345–360 nm (spectrometer UV-1800 (Shimadzu, Kyoto, Japan), Figure S1d), and blue–violet emission (photoluminescence) in the region 380–420 nm (spectrofluorimeter RF-600, Shimadzu, Kyoto, Japan, Figure S1c). The structure of dry ZnO NPs corresponded to the hexagonal structure of wurtzite according to Bragg scattering angles 2θ (100, 002, 101, 102, and 110) of powder X-Ray diffraction (XRD) patterns (X-ray diffractometer XRD-6000, Shimadzu, Kyoto, Japan, Figure S1a). The average powder size (D) of ZnO NPs was equal to 10–20 nm by the Scherrer equation.

### 4.3. Preparation and Composition of Anti-Burn Gels

#### 4.3.1. Oleogel ZnO NPs-BDP

Oleogel ZnO NPs-BDP preparation and properties were described in detail elsewhere [17]. Formulation of oleogel, %: ZnO NPs (5.0), BDP (1.0), sunflower oil up to 100.0.

#### 4.3.2. Hydrogel ZnO NPs-B—Lavender and Hydrogel ZnO NPs-BDP—Lavender

Hydrophilic phase A was prepared from a 2.1% aqueous suspension of hydroxyethyl cellulose (HEC) sonicated at 44 kHz for 3 min to a clear hydrogel. Lipophilic phase B was prepared as follows. First, surfactants—dipoly(hydroxy)stearate (PEG-30) and glyceryl stearate—were melted at a mass ratio of PEG-30:GS 1.5:1 by sonication at 44 kHz in a water bath at 55 °C for 10–15 min. Then, a homogenic betulin dispersion (2.6 or 1.6%) and BDP dispersion (1.0% in Sample 5, Table 4) in lavender oil was added, then a ZnO NPs (2.6%) was added with vigorous stirring at room temperature. Lipophilic phase B with vigorous stirring in a water bath at 55 °C was added into hydrophilic phase A and sonicated up to white thixotropic hydrogel formation.

#### 4.3.3. Oleogel ZnO NPs-BDP—Thymol-Lavender and Oleogel ZnO NPs-B—Thymol-Lavender

We successively mix betulin (13 or 12%) and BDP (0 or 1%), lavender oil (5%), thymol (5%) on an ultrasonic homogenizer at 50 °C (dispersion A). Then, the mixture of glyceryl stearate and ZnO NPs (6%) at 70 °C in sunflower oil (up to 100%) was added to dispersion A and mixed until a thick white thixotropic oleogel is formed.

### 4.4. Photoluminescence Analysis

Fluorescent spectra were obtained using spectrofluorometer RF-600 (Shimadzu, Kyoto, Japan) at an excitation wavelength 330 nm in the field 350–600 nm in a 10 mm-thick cuvette.

### 4.5. GL Chromatographical Analysis

Lavender oil was analyzed after transesterification, according to [52]. Fatty acid methyl esters were analyzed on a Chemito GC 8610 chromatograph equipped with a flame ionization detector and a BP x 70 capillary column (50 mm × 0.32 mm × 0.25 mm films); the temperature detector was equal to 260 °C with a flow rate of 0. 3 mL/min. The injector’s temperature was equal to 240 °C. Nitrogen (99.95% purity) was used as the carrier gas; the peaks were identified by comparing the retention time with that of the standards analyzed under the same conditions.

### 4.6. Viscosity Estimation

Viscosity of dosage forms was analyzed by viscometer Brookfield DV2T with spindle 64 (AMETEK Brookfield, Middleboro, MA, USA) equipped with a thermostat (Liquid low-temperature thermostat KRIO-VT-01; Termeks LLC, Tomsk, Russia) to maintain a temperature of 25 °C.

### 4.7. Specific Area Estimation

The specific surface area of powder materials was determined by the method of static vacuum volumetry (analyzer of surface area, micropore size, chemisorption “Autosorb iQ C” (Quantachrome Instruments, FL, USA). Before measurements, the samples were degassed under a dynamic vacuum (base pressure = 1.33 × 10 Pa) at a temperature of 120 °C for 3 h. The specific surface area of the powder was estimated by the Brunauer–Emmett–Teller (BET) method using data taken in the range 0.05 < p/po <0.35.

### 4.8. Surface Charge and Dynamic Light Scattering Measurements

Surface charge and average hydrodynamic diameter of the ZnO NPs were measured using NanoBrook Omni (Brookhaven Instruments, NY, USA). Suspensions with a solid loading of 0.00625–0.02500% were prepared in the presence (5 × 10^−4^ M) of BDP in different mediums and were allowed to equilibrate for 24 h to reach the steady-state. Zeta potential was determined by phase analysis electrophoretic light scattering (PALS). The Smoluchowski model was used to convert the electrophoretic mobility values to the zeta potential values.

The hydrodynamic diameter of ZnO nanoparticles was determined by dynamic light scattering (DLS) in the mode of multimodal analysis of the correlation function. The measurements were carried out at 25 ± 0.1 °C at an angle of 90 ° in the range from 0.1 to 5000 nm in polystyrene cuvettes (1 cm). The accumulation time of the correlation function was 180 sec (*n* = 10).

### 4.9. Elemental Analysis

Elemental Analysis was performed by ICP-AES, AAS, and complexometric titration.

ICP-AES analysis was carried out using inductively coupled plasma atomic emission spectrometer Prodigy High Dispersion ICP (Teledyne Leeman Labs., Hudson, NH, USA).

Elemental analysis was carried out using atomic absorption spectrophotometer AA-7000 (Shimadzu, Kyoto, Japan) at 213.9 nm. The samples were pretreated with HNO3:HClO4 (6:1) to leach out zinc metal. 

Complexometric titration was performed in accordance with the European Pharmacopoeia (2.5.11).

### 4.10. SEM Analysis

The morphology of the samples was obtained by scanning electron microscopy (SEM) on JSM-IT300LV (JEOL, Tokyo, Japan) with the electron probe diameter of about 5 nm and probe current below 0.5 nA (operating voltage 20 kV). The study of the sample surface topography was performed using the low-energy secondary electrons and backscattered electrons under low vacuum mode to eliminate charging.

### 4.11. Biological Activity

Male Wistar rats (150–200 g) were involved in the study. The animals were purchased from the Animal Breeding Facilities “Andreevka” Federal State Budgetary Institution of Science “Scientific Center for Biomedical Technologies” of the Federal Medical and Biological Agency (Andreevka, Moscow region, Russia). All procedures for maintenance and sacrifice (care and use) of animals were carried out according to the criteria outlined by European Convention ET/S 129, 1986 and directives 86/609 ESC. The animals were handled humanely, kept in plastic suspended cages, and placed in a well-ventilated and hygienic rat house under suitable conditions of room temperature (27 ± 2 °C) and humidity. They were given food and water ad libitum and subjected to a natural photoperiod of 12 h light and 12 h dark cycle. The animals were allowed two weeks of acclimatization before the commencement of all animal model experiments in the study.

All blood taking and withdrawal of animals out of experiment were performed under anesthesia, with all efforts being made to minimize suffering.

The study as presented was approved by the Local Ethics Committee of Privolzhsky Research Medical University, Russian Federation (protocol No. 1 from 18 January 2021; protocol No. 2 from 20 February 2016).

#### 4.11.1. Modeling of Thermal Burns in Animals

The surface of the animal’s back was burned using electromagnetic radiation from an infrared soldering station YaXunXY865D following the requirements of Good Laboratory Practice for experimental modeling of thermal burns in laboratory animals (CFR, Washington: Government Printing Office, 2009). We used the regime that causes thermal burns of the deep second degree. The distance of the infrared heater from the animal’s skin was 15 mm, the temperature on the skin in the heating zone was 60 °C, the heating duration was 23 s, the power was 100 W. Under these conditions, infrared radiation penetrates to a depth of 3–5 mm [53,54]. Standard thermal burns had an area of 14.0 ± 0.5 cm^2^.

#### 4.11.2. Biological Activity In Vitro

Biological analysis in vitro was performed using blood stabilized with sodium citrate. Erythrocytes were washed twice with 0.9% NaCl by centrifugation for 10 min at 1600 g. The intensity of lipid peroxidation (LPO) was estimated by the MDA level in plasma and erythrocytes following the methods by Uchiyama and Mihara [55]. Superoxide dismutase activity (EC 1.15.1.1) was measured in erythrocytes using inhibition of adrenaline auto-oxidation [56]. Catalase activity (EC 1.11.1.6) was determined by spectrophotometry based on the decomposition of hydrogen peroxide by the catalase [57]. Glutathione reductase activity (EC 1.8.1.7) was studied by spectrophotometry based on the oxidized glutathione reduction [58]. The activity of glucose-6-phosphate dehydrogenase (EC 1.1.1.49) was determined in hemolysate of erythrocytes by spectrophotometry based on glucose-6-phosphate oxidation to the phosphoglucolactone with the formation of reduced nicotinamide adenine dinucleotide phosphate (NADPH) [59]. The energy metabolism in erythrocytes was studied using the catalytic activity of LDH (LDH, EC 1.1.1.27), directly (LDHdirect, substrate—50 mM sodium lactate) and in reverse (LDHreverse, substrate—23 mM sodium pyruvate) reactions [60]. The activity of aldehyde dehydrogenase (EC 1.2.1.3) was estimated spectrophotometrically in accordance with previous methods [61]. The specific activity of the enzymes was calculated from the protein concentration analyzed by the modified Lowry method [62].

### 4.12. Quantum-Chemical Calculations

Quantum-chemical calculations of the sizes of betulin dimers were performed using the HyperChem program (version 7.52). The MM + field was chosen as the force field of molecular mechanics (potential function of calculations). To optimize the geometry and minimize the system’s energy, the Polak–Ribiere algorithm was chosen [63]. We used the DFT method (B3-lyp) to calculate the charge density.

### 4.13. Statistical Analysis

Statistical data processing was performed by the software (Statistica 6.0 (StatSoft Inc., Tulsa, OK, USA)). The normality of a distribution of results was shown using the Shapiro–Wilk test. The significance of differences between groups was assessed using Student’s t-test and one-way analysis of variance (ANOVA). The differences were considered statistically significant at *p* < 0.05.

## 5. Conclusions

In this work, the possibility of using modified ZnO NPs as an active pharmaceutical substance for the preparation of both hydrophilic and oleophilic dosage forms for the treatment of thermal burns has been studied. We proposed to use terpenes of essential oils (thyme and lavender) as a component and modifier of ZnO NPs, and triterpenoids of the lupane group (betulin and BDP) which have proven themselves as an anti-inflammatory agent in the treatment of burns. The presence of BDP leads in a polar medium to a 20-fold increase in the hydrodynamic radius of ZnO NPs, which creates the prerequisites for creating hydrophilic gel-like dispersions. The additional introduction of the components of essential oils—thymol, linalool, and other monoterpenes, contributes to reducing inflammation in the burn wound and the solubilization of betulin and BDP in dispersions. The resulting gel-like dispersions exhibit thixotropic properties, established by the presence of a hysteresis loop on rheograms, which makes them convenient to apply to a burn wound.

The advantage of hydrophilic gel-like dispersions is the possibility of forming zinc ions from ZnO NPs in a weakly acidic polar medium created by BDP and their penetration into the skin. Penetration and penetration of zinc ions into the systemic circulation along with terpenes contributes to a more efficient restoration of redox balance of co-enzyme pairs NAD+/NADH and NADP+/NADPH, as well as the activity of SOD, catalase, AlDH in erythrocytes, LDH, GR, and G6PDH in the treatment of burns in rats. 

Thus, our studies have shown that improving the energy metabolism and the restoration of the redox balance by treatment with ZnO NPs modified by terpenoids as oleophilic and hydrophilic gel-like dispersions lead on day 21 to an improvement in the healing of burns.

## Figures and Tables

**Table 1:** Data of PXRD patterns of ZnO NPs modified by lavender oil and BDP (Figure 2).

	ZnO NPs-Lavender Oil			ZnO NPs-BDP [17]		
Peak	101	002	100	101	002	100
β‘, 2θ	0.595	0.476	0.595	0.714	0.476	0.714
β, rad	0.0103	0.0083	0.0103	0.0125	0.0083	0.0125
2θ, °	31.68	34.34	36.42	31.76	34.36	36.22
Cosθ	0.959	0.955	0.950	0.959	0.954	0.950
D, nm	17.3	17.5	14.0	11.6	17.5	11.7

**Table 2:** Zeta potential of ZnO NPs dispersions at pH 7.3 in various media.

№	ZnO NPs, %	Medium	Zeta Potential, mV
1	0.00625	1.25 × 10^−4^ M citric acid in ethanol: water (1:1)	−6.47 ± 1.10
2	0.00625	1.25 × 10^−4^ M BDP in ethanol	−1.49 ± 1.81
3	0.025	5 × 10^−4^ M sodium salt of BDP in ethanol: water (1:1)	−7.09 ± 0.25
4	0.025	5 × 10^−4^ M BDP in ethanol	−6.51 ± 0.60
5	0.025	ethanol: water (1:1)	+15.90
6	0.025 ^1^	1 × 10^−3^ M BDP in ethanol	−41.20

^1^ ZnO NPs pre-modified with BDP [25].

**Table 3:** Data of the FTIR spectra of betulin, thymol, and a mixture of betulin: thymol (1:1).

ν,cm^−1^	-OH 3200–3600	-C-O 1000–1100	=CH_2_ 800–890
Betulin	3380	1028	881
Thymol	3280	1091	806
Betulin: Thymol mixture (1:1)	3380, 3280, 3550	1044	804

**Table 4:** Composition (%) of gel-like dispersions containing of ZnO NPs and terpenoids.

Number	Dispersion	ZnO NPs	B	BDP	Surfactant	Excipients	Medium
1	Oleo ZnO NPs-BDP [17]	5.0	10.0	1.0	-	Ascorbic acid 0.1 α-tocopherol acetate 0.1	Sunflower oil up to 100
2	Oleo ZnO NPs-BDP—lavender	6.0	12.0	1.0	GS—1.0	Thymol 2.0 Lavender oil 2.0	Sunflower oil up to 100
3	Oleo ZnO NPs-B—lavender	6.0	13.0	-	GS—1.0	Thymol 2.0 Lavender oil 2.0	Sunflower oil up to 100
4	Hydro ZnO NPs-B	4.7	6.0	-	PEG30—3.7 GS—2.7	HEC—2.1 Lavender oil 2.0	distilled water up to 100
5	Hydro ZnO NPs-BDP	4.7	5.0	1.0	PEG30—3.7 GS—2.7	HEC—2.1 Lavender oil 2.0	distilled water up to 100

Note: GS—glyceryl stearate; PEG30—polyethylene glycol; HEC—hydroxy ethyl cellulose.

**Table 5:** Assay of ZnO NPs in dispersions (*n* = 3).

Dispersion	D, nm	ZnO, %		
		Added	Founded	
			Complexonometry	AAS
Oleo ZnO NPs-BDP—thymol-lavender	15.74 ± 0.93	6.0	5.98 ± 0.67	5.87 ± 0.31
Oleo ZnO NPs-B—thymol-lavender	16.42 ± 2.23	6.0	5.91 ± 0.91	5.85 ± 0.83
Hydro ZnO NPs-B—lavender	16.90 ± 4.12	4.7	4.71 ± 0.13	4.68 ± 0.43
Hydro ZnO NPs-BDP—lavender	15.89 ± 4.33	4.7	4.65 ± 0.20	4.61 ± 0.35

**Table 6:** Wound area changes during treatment with hydrophilic and oleophilic gel-like dispersions.

Group	Wound Area, cm^2^		
	0 Day	10 Day	21 Day
Burnt untreated	21.746 ± 0.612	20.127 ± 0.230	16.184 ± 0.971
Oleo ZnO NPs-BDP	21.599 ± 0.628	19.897 ± 0.313	12.825 ± 0.311
Oleo ZnO NPs-BDP—thymol-lavender	22.620 ± 0.199	18.384 ± 1.321	10.028 ± 0.224
Oleo ZnO NPs-B—thymol-lavender	23.714 ± 0.392	19.329 ± 0.968	11.519 ± 0.120
Hydro ZnO NPs-B—lavender	22.783 ± 0.198	18.635 ± 0.457	11.689 ± 0.672
Hydro ZnO NPs-BDP—lavender	21.459 ± 0231	18.058 ± 0.391	10.818 ± 0.973

**Table 7:** Glucose-6-phosphate dehydrogenase activity during treatment with oleophilic and hydrophilic gel-like drugs (% of control), *n* = 3, *p* <0.001.

τ, Day	G6PDH Activity, % of Control ^1^		
	Burnt (Untreated)	Oleo ZnO NPs-BDP—Thymol-Lavender	Hydro ZnO NPs-BDP—Lavender
3	79.52 ± 2.17	100.93 ± 3.51	146.01 ± 1.68
7	90.14 ± 1.57	117.72 ± 1.96	152.80 ± 2.86
10	104.02 ± 1.92	128.02 ± 3.37	165.21 ± 3.63
21	N/a	138.12 ± 3.60	187.48 ± 4.22

^1^ 29.024 ± 0.761 nmol NADPH min^−1^ mg protein^−1^ is taken as 100%.

**Table 8:** Lactate dehydrogenase activity in the direct and reverse reactions during treatment with oleophilic and hydrophilic gel-like drugs (% of control), *n* = 3, *p* <0.001.

Enzyme	τ, Day	LDH Activity, % of Control ^1^		
		Burnt (Untreated)	Oleo ZnO NPs-BDP—Thymol-Lavender	Hydro ZnO NPs-BDP—Lavender
**LDH_direct_**	3	73.25 ± 1.61	89.15 ± 0.98	107.49 ± 2.31
	7	101.32 ± 0.82	115.06 ± 3.18	118.39 ± 0.60
	10	134.14 ± 1.58	141.43 ± 1.44	125.08 ± 2.11
	21	N/a	120.93 ± 1.62	134.28 ± 0.61
**LDH_reverse_**	3	87.23 ± 0.92	150.59 ± 2.03	172.10 ± 1.83
	7	92.38 ± 1.96	120.19 ± 1.85	153.72 ± 0.29
	10	102.01 ± 1.36	125.82 ± 0.45	107.42 ± 1.99
	21	N/a	127.18 ± 2.27	100.93 ± 0.82

^1^ LDH_direct_ 100%—20.215 ± 0.482 nmol NADH min^−1^ mg protein^−1^; LDH_reverse_ 100%—124.173 ± 3.892 nmol NADH min^−1^ mg protein^−1^.

**Table 9:** Glutathione reductase activity during treatment with oleophilic and hydrophilic gel-like dosage forms (% of control), *n* = 3, *p* <0.001.

τ, Day	GR Activity, % of Control ^1^		
	Burnt (Untreated)	Oleo ZnO NPs-BDP—Thymol-Lavender	Hydro ZnO NPs-BDP—Lavender
3	58.23 ± 0.94	80.73 ± 1.80	101.23 ± 5.25
7	67.05 ± 0.58	87.04 ± 5.71	104.09 ± 2.65
10	73.77 ± 1.43	91.62 ± 3.03	106.38 ± 1.02
21		120.27 ± 2.95	121.87 ± 3.01

^1^ 100%—95.833 ± 2.324 nmol NADPH min^−1^ mg protein^−1^ is taken as 100%.

**Table 10:** MDA level in plasma and blood erythrocytes (μmol/L) during treatment with oleophilic and hydrophilic gel-like dosage forms (% of control), *n* = 3, *p* <0.001.

	τ, Day	MDA Level, % of Control		
		Burnt (Untreated)	Oleo ZnO NPs-BDP—Thymol-Lavender	Hydro ZnO NPs-BDP—Lavender
**MDA_pl_**	3	240.32 ± 6.22	231.34 ± 3.72	169.82 ± 2.99
	7	180.48 ± 3.83	152.21 ± 8.60	143.73 ± 3.92
	10	150.62 ± 4.09	124.83 ± 5.34	121.82 ± 7.28
	21	N/a	112.04 ± 5.22	85.48 ± 5.27
**MDA_er_**	3	152.62 ± 5.38	153.81 ± 4.94	126.13 ± 8.17
	7	147.24 ± 4.24	122.25 ± 6.37	113.67 ± 7.51
	10	140.72 ± 8.12	103.78 ± 4.32	96.14 ± 8.11
	21	N/a	86.42 ± 4.92	84.66 ± 4.57

**Table 11:** SOD activity during treatment with oleophilic and hydrophilic gel-like dosage forms (% of control), *n* = 3, *p* <0.001.

τ, Day	SOD Activity, % of Control ^1^		
	Burnt (Untreated)	Oleo ZnO NPs-BDP—Thymol-Lavender	Hydro ZnO NPs-BDP—Lavender
3	61.54 ± 0.82	83.63 ± 3.61	102.26 ± 6.20
7	48.27 ± 0.62	102.24 ± 4.61	121.73 ± 7.72
10	54.21 ± 2.01	107.71 ± 1.73	123.28 ± 2.67
21		113.24 ± 5.26	126.91 ± 7.19

^1^ 1048.419 ± 16.820 Ru mg protein^−1^ is taken as 100%.

**Table 12:** Catalase activity during treatment with oleophilic and hydrophilic gel-like dosage forms (% of control), *n* = 3, *p* <0.001.

τ, Day	Catalase Activity, % of Control ^1^		
	Burnt (Untreated)	Oleo ZnO NPs-BDP—Thymol-Lavender	Hydro ZnO NPs-BDP—Lavender
3	43.82 ± 3.10	52.92 ± 2.87	56.28 ± 2.82
7	52.37 ± 0.82	61.78 ± 3.16	75.60 ± 7.28
10	61.72 ± 1.78	83.48 ± 3.72	92.03 ± 4.82
21	-	118.34 ± 2.02	120.82 ± 5.22

^1^ 42.712 ± 0.822 μmol H_2_O_2_ min^−1^ mg protein^−1^ is taken as 100%.

**Table 13:** Aldehyde dehydrogenase activity during treatment with oleophilic and hydrophilic gel-like dosage forms (% of control), *n* = 3, *p* <0.001.

τ, Day	AlDH Activity, % of Control ^1^		
	Burnt (Untreated)	Oleo ZnO NPs-BDP—Thymol-Lavender	Hydro ZnO NPs-BDP—Lavender
3	51.21 ± 0.88	146.56 ± 2.92	149.82 ± 4.62
7	50.76 ± 2.82	142.53 ± 6.82	132.48 ± 4.51
10	56.37 ± 2.37	134.82 ± 3.37	121.32 ± 6.70
21		106.82 ± 5.40	95.06 ± 2.42

^1^ 23.518 ± 0.769 nmol NADH min^−1^ mg protein^−1^ is taken as 100%.

## Data Availability

Not applicable.

## Supplementary Materials

The following are available online at https://www.mdpi.com/article/10.3390/ph14060492/s1, Figure S1: Physicochemical properties of ZnO NPs: PXRD pattern, insert—standard JCPDS pattern for ZnO (file 036-1451) (a); FTIR-spectrum (b); PL spectrum of 27.2 mg% ethanolic dispersion of ZnO NPs, insert—digital image of dispersion under UV-lamp (λ_ex_ = 254 nm) (c); UV spectrum of ZnO NPs in ethanol, 13.6 mg/%, blanc—ethanol (d), Figure S2: SEM images of ZnO NPs (a) and ZnO NPs modified by BDP (b), ×30,000, Figure S3: FTIR spectra of B (a), ZnO NPs modified by B (b), BDP (c), ZnO NPs modified by BDP (d), Figure S4: GL chromatograms and composition of lavender oil before transesterification (a) and after methylation (b), Figure S5: GL chromatogram and composition of thymol, Figure S6: Geometric visualization of BDP as a component of inclusion complexes formed by two and four molecules. Quantum-chemical calculations were provided by HyperChem 8.0, Hypercube Inc, Gainesville, FL, USA (semiempirical method AM1). Size of the void between BDP molecules is 6 Å for dimer and 10 Å for tetramer, Figure S7: UV spectra of samples of ZnO NPs dispersions (27 mg/%) in ethanol 95%: ZnO NPs (1); ZnO NPs pretreated with: alcohol solutions 2% lavender oil (2); alcohol solutions of 1.4% thymol (3); 2% lavender oil and 0.30% betulin diphosphate (4); 2% lavender oil and 0.22% betulin (5), Figure S8: HPL chromatograms of initial solutions of betulin and betulin diphosphate (insert—HPL chromatograms after sorption on the ZnO NPs surface), Figure S9: Digital images of gel-like dispersions (Oleo ZnO NPs-BDP–thymol-lavender and Hydro ZnO NPs-BDP–lavender) under daylight and UV-lamp (λ_ex_ = 254 nm), Figure S10: Wound state under treatment by Oleo ZnO NPs-BDP–thymol-lavender and by Hydro ZnO NPs-BDP–lavender for 3, 10 and 21 days, Figure S11: Histological images under treatment by Oleo ZnO NPs-BDP–thymol-lavender and by Hydro ZnO NPs-BDP–lavender for 10 and 21 days.
