# Supplementary material for: Zinc Oxide Nanoparticles Protected with Terpenoids as a Substance in Redox Imbalance Normalization in Burns
Source: Pharmaceuticals (Basel). 2021 May 21;14(6):492. doi: 10.3390/ph14060492 (PMC8224349; doi:10.3390/ph14060492)
Supplement: Supplementary file 1 [file pharmaceuticals-14-00492-s001.zip › pharmaceuticals-1200294-supplementary.pdf]

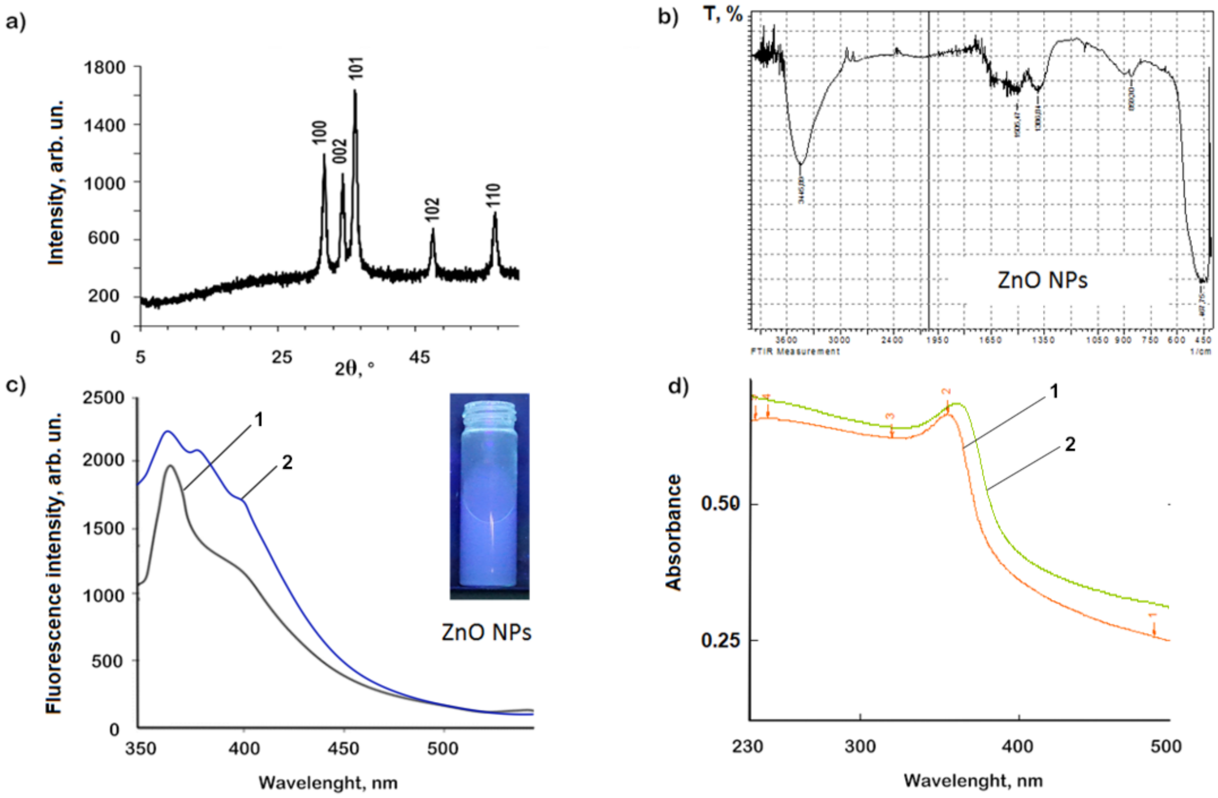

Figure S1. Physicochemical properties of ZnO NPs: PXRD pattern (a); FTIR-spectrum (b); PL spectrum of 27.2 mg% ethanolic dispersion of ZnO NPs, insert – digital image of dispersion under UV-lamp (λ_{ex} = 254 nm) (c); UV spectrum of ZnO NPs in ethanol, 13.6 mg/%, blanc – ethanol (d). Note: curve 1 and curve 2 (Figure S1c,d) indicate spectra recorded in this paper (curve 2), and our paper [17] published early (curve 1) of ZnO NPs obtained by the same methods.

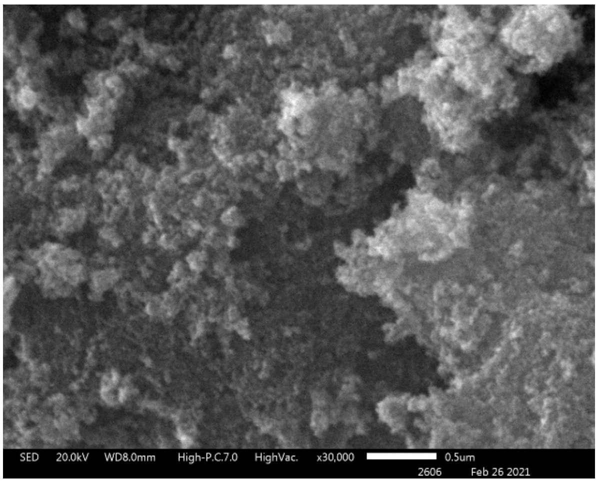

(**a**)

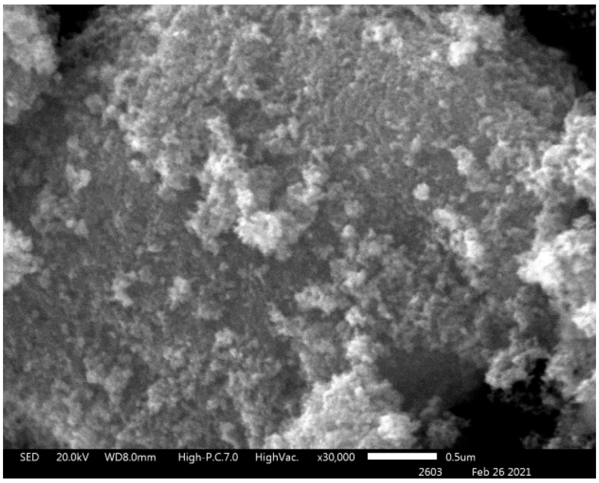

(**b**)

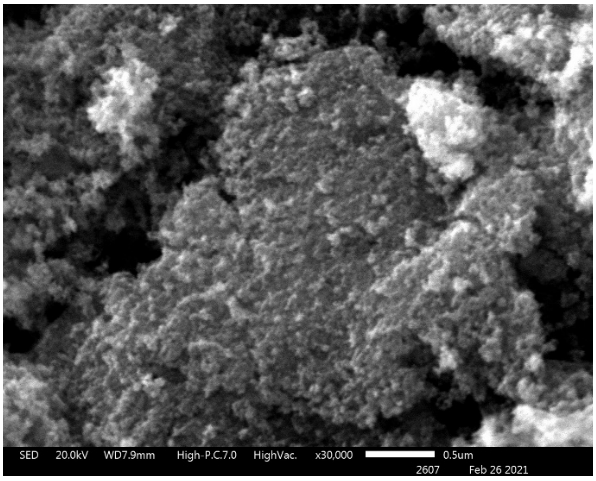

(c)

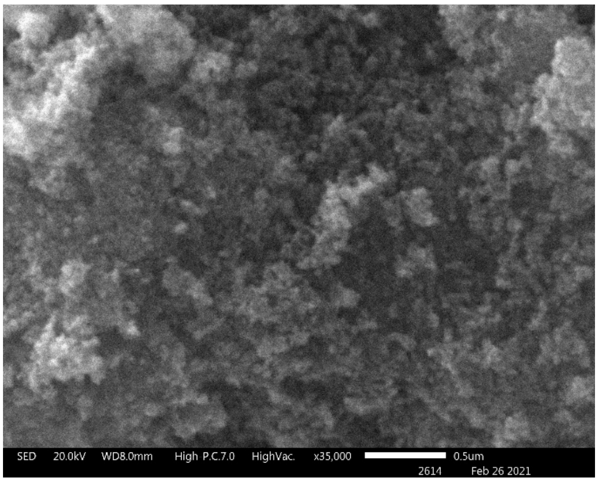

(d)

Figure S2. Comparison of SEM images of ZnO NPs (a, c) with SEM images of ZnO NPs modified by BDP (b, d), ×30 000. Note: Figures S2c, d indicate SEM images recorded in this paper. Figures S2a, b were obtained in our paper [25] early.

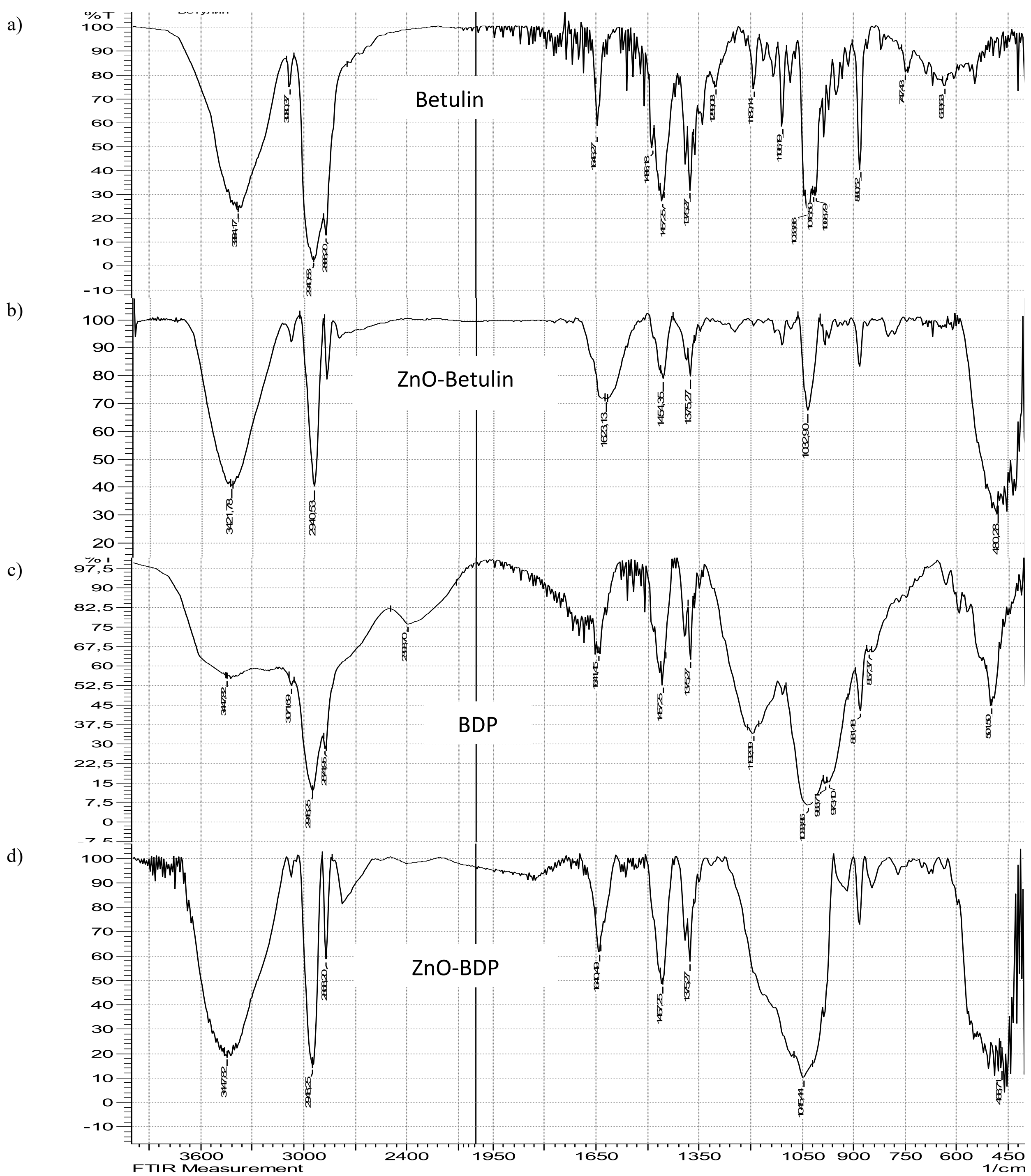

Sample	3450-3350 cm^{-1} ν (OH)	2900-2800 cm^{-1} ν (CH, CH_2, CH_3)	1800-1600 cm^{-1} δ (P-OH), ρ (PO-H), ν (CH_2), δ (H_2O)	1200-900 cm^{-1} ν (HC-OH, H_2C-OH)	800-400 cm^{-1}	
					δ (O-P-O) ν O-PO_3 υ (P=O)	ν ZnO NPs
Betulin	3384	2941, 2868	1646	1050, 1034	-	-
ZnO-Betulin	3422	2941, 2870	1623	1033	-	480
BDP	3448	2948, 2875	1641	1034	502	-
ZnO-BDP	3448	2948, 2868	1640	1045	-	469

Figure S3. FTIR spectra of Betulin (a), ZnO NPs modified by Betulin (b), BDP (c), ZnO NPs modified by BDP (d).

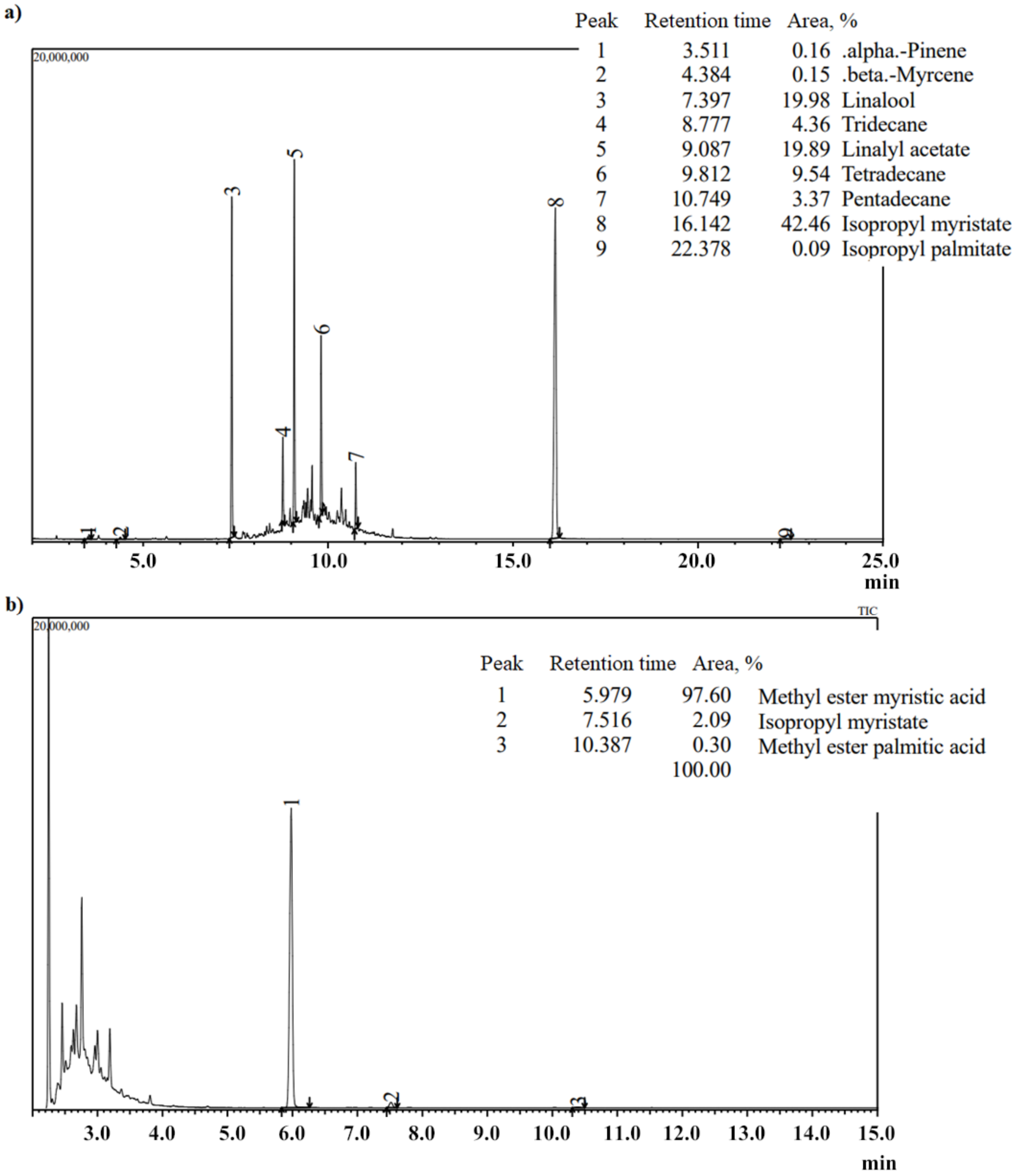

Figure S4. GL chromatograms and composition of lavender oil before transesterification (a) and after methylation (b).

Peak	Retention time	Area, %	
1	3.529	3.21	.alpha.-Pinene
2	3.856	0.42	Camphene
3	4.011	0.15	Cyclohexane, 1-methyl-4-(1-methylethyl)-, ci
4	4.337	2.78	.beta.-Pinene
5	4.404	1.58	.beta.-Myrcene
6	5.287	1.33	D-Limonene
7	5.577	39.63	o-Cymene
8	5.660	2.13	Eucalyptol
9	6.392	0.26	Cyclohexene, 3-methyl-6-(1-methylethyliden
10	7.409	9.11	Linalool
11	8.314	0.20	Cyclohexanol, 1-methyl-4-(1-methylethenyl)-
12	8.493	0.23	(+)-2-Bornanone
13	8.750	1.15	endo-Borneol
14	8.866	2.34	.alpha.-Terpineol
15	8.919	0.56	Cyclohexanol, 1-methyl-4-(1-methylethylider
16	10.592	31.29	Thymol
17	10.726	3.62	Bicyclo[7.2.0]undec-4-ene, 4,11,11-trimethyl
		100.00	

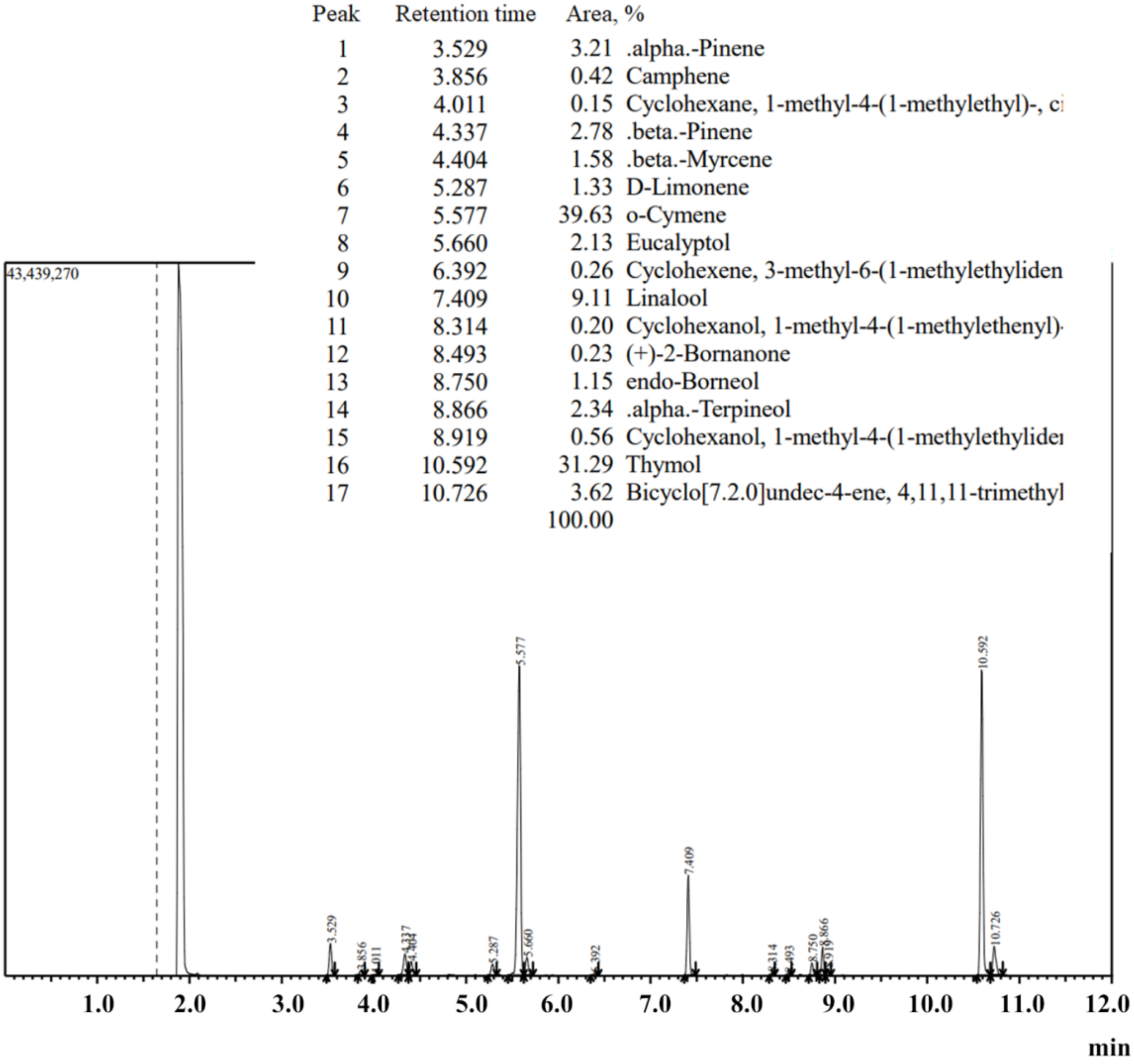

Figure S5. GL chromatogram and composition of thymol.

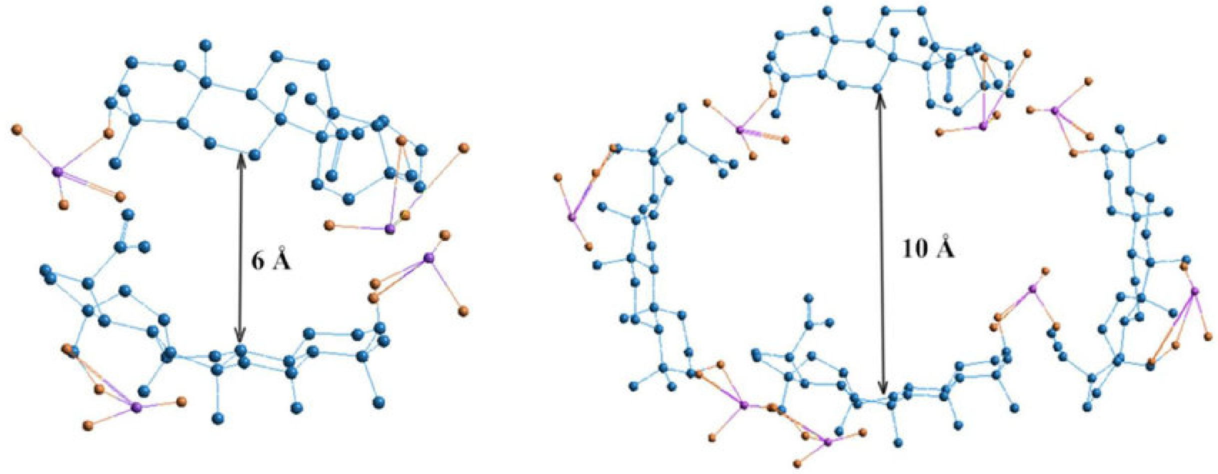

Figure S6. Geometric visualization of BDP as a component of inclusion complexes formed by two and four molecules. Quantum-chemical calculations were provided by HyperChem 8.0, Hypercube Inc, Gainesville, FL, USA (semiempirical method AM1). Size of the void between BDP molecules is 6 Å for dimer and 10 Å for tetramer.

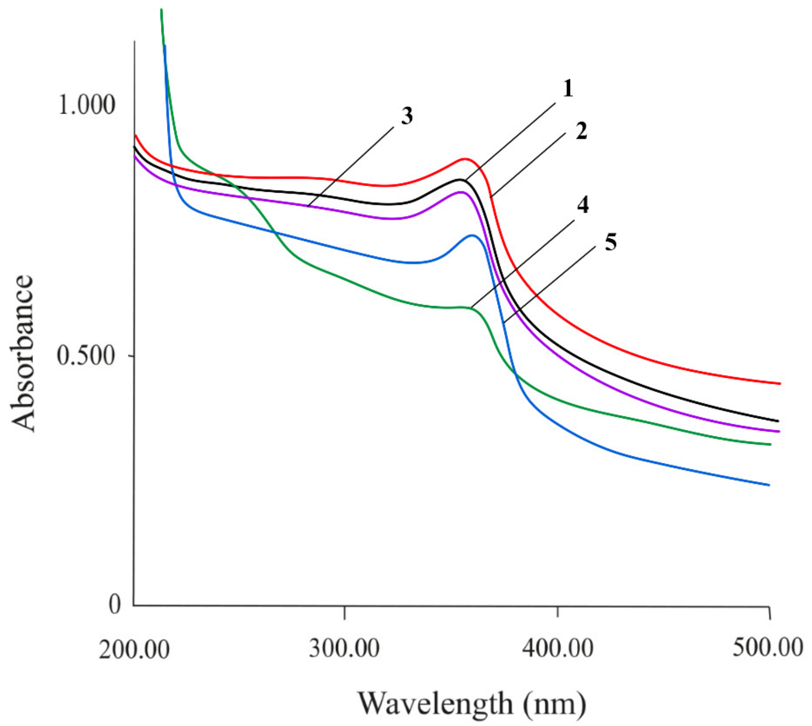

Figure S7. UV-spectra of samples of ZnO NPs dispersions (27 mg/%) in ethanol 95%: ZnO NPs (1); ZnO NPs pretreated with: alcohol solutions 2% lavender oil (2); alcohol solutions of 1.4% thymol (3); 2% lavender oil and 0.30% betulin diphosphate (4); 2% lavender oil and 0.22% betulin (5).

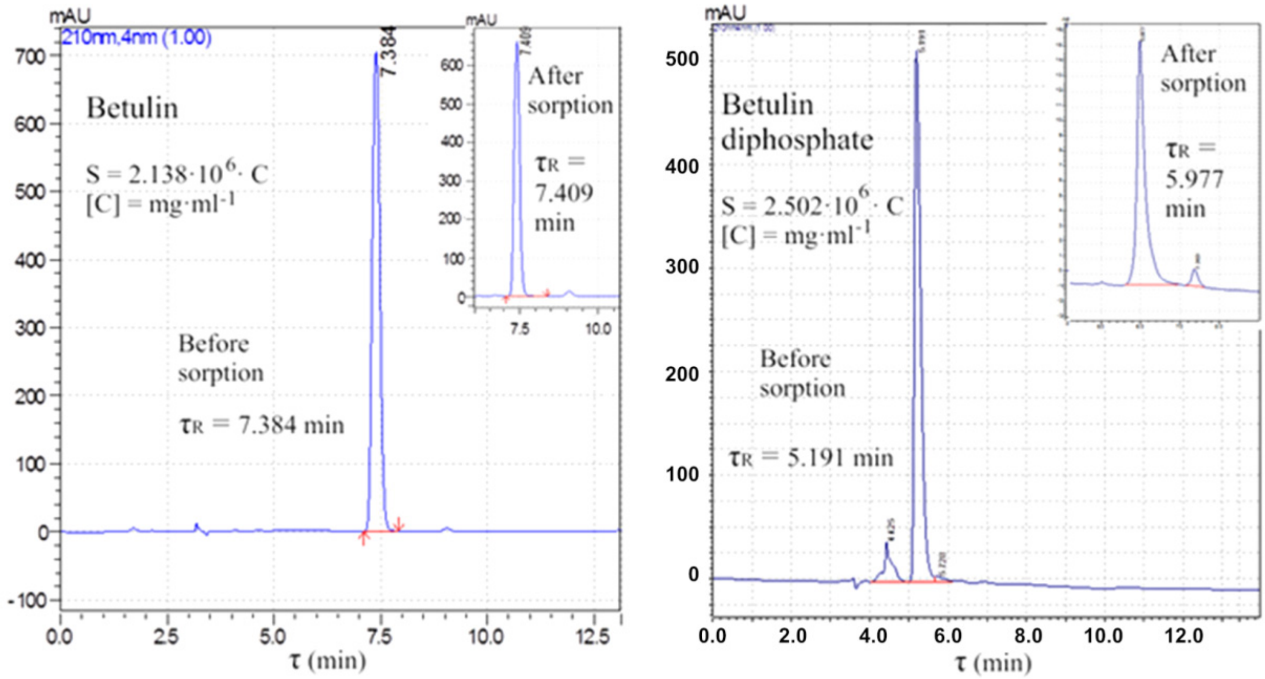

Figure S8. HPL chromatograms of initial solutions of betulin and betulin diphosphate (insert – HPL chromatograms after sorption on the ZnO NPs surface).

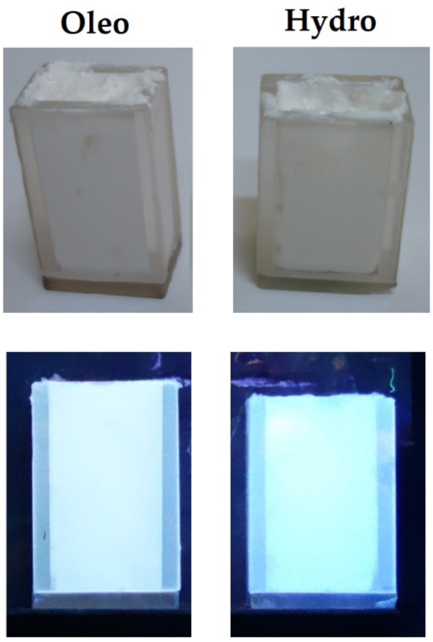

Figure S9. Digital images of gel-like dispersions (Oleo ZnO NPs-BDP–thymol-lavender and Hydro ZnO NPs-BDP–lavender) under daylight and UV-lamp (λ_{ex} = 254 nm).

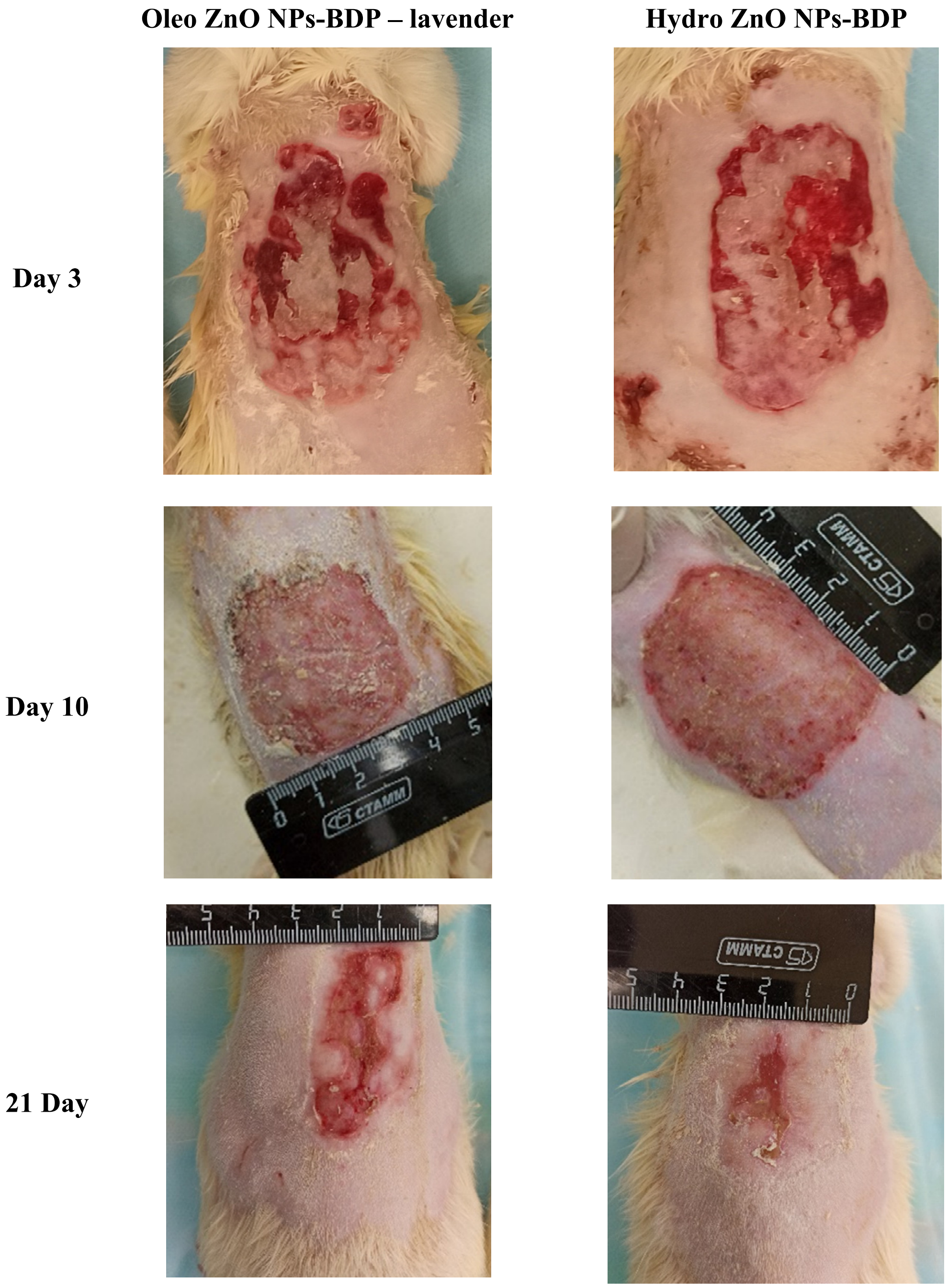

Figure S10. Wound state under treatment by Oleo ZnO NPs-BDP-thymol-lavender and by Hydro ZnO NPs-BDP-lavender for 3, 10 and 21 days.

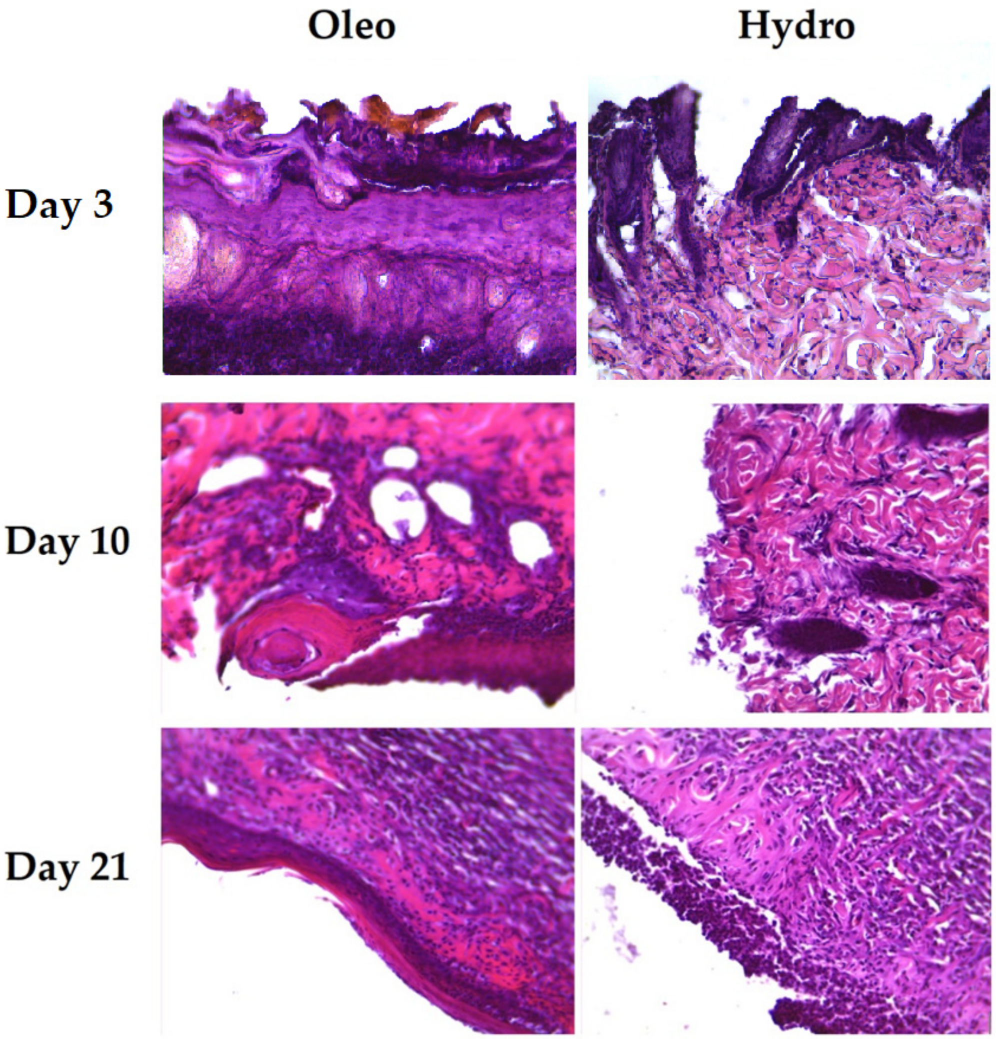

Figure S11. Histological images under treatment by Oleo ZnO NPs-BDP-thymol-lavender and by Hydro ZnO NPs-BDP-lavender for 10 and 21 days.
